# UiO‐Based Mixed Matrix Membranes for Efficient CO_2_ Separations

**DOI:** 10.1002/cplu.202500151

**Published:** 2025-05-29

**Authors:** Lamprini G. Boutsika, Christos Tampaxis, Kyriaki G. Papadokostaki, Merope Sanopoulou, Georgia Charalambopoulou, Ioannis Bratsos, Theodore Steriotis

**Affiliations:** ^1^ Institute of Nanoscience and Nanotechnology National Centre for Scientific Research “Demokritos” Ag. Paraskevi Attikis 15341 Greece; ^2^ Institute of Nuclear & Radiological Sciences & Technology Energy & Safety National Centre for Scientific Research “Demokritos” Ag. Paraskevi Attikis 15341 Greece

**Keywords:** adsorptions, metal‐organic frameworks, mixed matrix membranes, Pebax, permeabilities

## Abstract

Mixed matrix membranes (MMMs) containing UiO‐type metal‐organic frameworks (MOFs) have shown excellent potential for CO_2_ separation processes due to their unique permeability and selectivity properties. However, while the performance of MOF‐based MMMs has been widely studied, the effect of structural defects and polymer‐filler compatibility are not yet fully understood. In this work, the CO_2_ separation performance of Pebax MH1657‐based MMMs is systematically evaluated incorporating 5–20 wt% of Zr‐based MOF, including UiO‐66, UiO‐67, and two defect‐engineered UiO‐66 analogues, featuring extended linker (UiO‐66_A) or cluster (UiO‐66_F) vacancies. The structural, morphological, and thermal properties of the membranes are thoroughly characterized, with emphasis on correlating these features with gas transport performance. Single‐gas permeation experiments (CO_2_, CH_4_, H_2_) reveal that incorporating UiO nanoparticles within the matrix consistently enhanced CO_2_ permeability, reaching 145 Barrer for the 20 wt% UiO‐66_F MMM, a 216.4% increase over the neat membrane. CO_2_/CH_4_ and CO_2_/H_2_ selectivities also improve upon increasing MOF loadings, with UiO‐66_F achieving values of 25 and 18, respectively. This study provides insights for designing high‐performance MMMs for CO_2_ separation applications, such as biogas upgrading and hydrogen purification.

## Graphical abstract

Pebax‐based mixed matrix membrane incorporating UiO‐type metal‐organic frameworks are developed for CO_2_ separations. Defect engineering and optimized dispersion enhanced CO_2_ sorption and transport pathways, significantly improving permeability while maintaining high selectivity over CH_4_ and H_2_. The optimized membranes offer a promising, balanced performance for applications such as biogas upgrading and hydrogen purification.

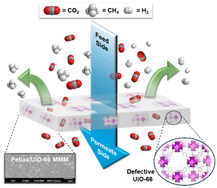

## 1 Introduction

The global push toward renewable energy sources has intensified in recent years due to the need to reduce dependence on fossil fuels and greenhouse gas emissions. Biogas plays a vital role in the transition to a more sustainable energy system, with projections indicating that the global demand for biogas and biomethane will increase by 30% between 2024 and 2030, reaching 2,270 PJ annually by 2030.^[^ ^1^ ^]^ Biogas is produced via the anaerobic decomposition of biomass, yielding a mixture primarily composed of CH_4_ and CO_2_. To upgrade biogas to bio‐methane, which is a renewable alternative to natural gas, CO_2_ must be removed, as it reduces the calorific value of biogas and causes pipeline corrosion.^[^ ^2^ ^]^ Similarly, in hydrogen production processes such as biomass gasification or steam reforming (e.g., of biogas), CO_2_/H_2_ separation is crucial for obtaining high‐purity hydrogen.^[^ ^3^ ^]^ Membrane separation has emerged as a promising technology for CO_2_ removal in such processes due to its simplicity, low operating costs, modularity, environmental friendliness, and energy efficiency compared to conventional separation methods.^[^ ^4^, ^5^, ^6^ ^]^

Polymeric membranes, known for their superior mechanical properties, thermal stability, and long‐term reliability, are widely used in CO_2_ separation. Some membranes, such as the broadly studied Pebax, are inherently CO_2_‐philic, while others are engineered to exhibit CO_2_‐philic properties. However, their gas separation performance is typically constrained by the trade‐off between permeability and selectivity, usually represented by the Robeson's upper bound.^[^ ^7^, ^8^ ^]^ To address this limitation, extensive research has been devoted to enhance the efficiency of polymeric membranes, aiming to develop materials with suitable performance that can compete with or surpass traditional separation solutions.^[^ ^9^ ^]^ Several strategies have been explored to overcome the permeability‐selectivity trade‐off, including polymer blends, cross‐linking, copolymerization, surface modification, and incorporation of functional fillers.^[^ ^10^, ^11^, ^12^, ^13^ ^]^ Among these attempts, mixed matrix membranes (MMMs), which are developed by incorporating inorganic fillers into polymer matrices, have in several cases revealed increased gas permeability and selectivity. Nevertheless, surpassing Robeson's upper bound remains a major challenge, which in the case of MMMs has stimulated the search for new advanced fillers with tailored properties to meet the specific requirements for CO_2_ separation. Recent efforts have focused on nanoporous materials, particularly metal‐organic frameworks (MOFs), which offer a unique combination of ultrahigh surface area, tunable pore size, notable thermal and chemical stability, and excellent gas adsorption properties. MOFs also offer the flexibility to tailor their structure and chemistry by selecting specific organic linkers and metal nodes, allowing for tunable interactions with the polymer matrix and as such they are among the most extensively studied filler materials for MMMs; over 50% of published research on MMMs for gas separation are focusing on MOF‐based membranes.^[^ ^14^ ^]^

The most suitable MOFs for the development of CO_2_ separating MMMs are CO_2_‐philic structures such as UiO‐type MOFs, and in particular UiO‐66 analogues, which are frequently used as fillers in MMMs due to their significant CO_2_ affinity, attributed to the —OH groups on the Zr cluster,^[^ ^15^, ^16^, ^17^ ^]^ coupled with their ultrahigh microporosity and remarkable chemical/thermal stability. Moreover, the UiO‐66 framework is highly versatile, as it can be readily functionalized or modified by introducing various groups, allowing precise tuning of pore architecture and adsorption properties.^[^ ^18^, ^19^ ^]^ Defect engineering, achieved by introducing areas with missing linkers or clusters into the UiO‐66 framework, can further expand its applicability by enabling control over pore size and chemical heterogeneity, which in turn has a profound effect on its CO_2_ adsorption capacity.^[^ ^16^, ^17^ ^]^

Several studies have demonstrated the effectiveness of UiO‐type MOFs in enhancing the performance of MMMs for gas separation processes. For instance, incorporation of 32 wt% UiO‐66 into Udel 3500‐P and Matrimid polymers improved H_2_/CH_4_ selectivity by 6.5 and 7.7%, respectively, while tripling the CO_2_ permeability, thus significantly enhancing the CO_2_/CH_4_ separation performance.^[^ ^20^ ^]^ In another study, UiO‐66 was integrated into 6FDA‐based (6FDA = (4,4′‐hexafluoroisopropylidene)diphthalic anhydride) co‐polyimides with loadings 4–23 wt%, resulting in CO_2_ permeability and CO_2_/CH_4_ selectivity improvements of 50–180% and 70–220%, respectively.^[^ ^21^ ^]^ A hollow defect‐engineered UiO‐66‐based MMM with 6 wt% loading demonstrated an increase of CO_2_ permeability and CO_2_/N_2_ selectivity by 129.5 and 242.3%, respectively, compared to neat Pebax‐2533 membrane.^[^ ^22^ ^]^ Similarly, a functionalized variant of UiO‐67 (namely UiO‐67 integrating 33% 2,2'‐bipyridine‐5,5'‐dicarboxylic acid), demonstrated significant enhancements in both permeability and selectivity when incorporated into Matrimid membranes, achieving up to a 350% increase in permeability and two times higher selectivity.^[^ ^23^ ^]^

Building on these advancements, the present work focuses on the development and comparative analysis of new composite membranes incorporating a series of Zr‐based MOF particles into Pebax MH1657 polymer matrices to enhance CO_2_ separation performance. Pebax, a commercial block copolymer composed of 40 wt% rigid polyamide (PA) and 60 wt% flexible polyethylene oxide (PEO) segments, is known for its effectiveness in separating CO_2_ from gas mixtures, mechanical flexibility, and compatibility with various fillers, making it a good candidate for gas separation applications. To systematically investigate the effects of MOF porosity and structural defects, as well as of polymer–filler interactions on membrane performance, varying loadings (5, 10, and 20 wt%) of different UiO‐type MOFs were incorporated into the polymer matrix. The study focused on using UiO‐66 and its expanded analogue UiO‐67, which features larger pores, to assess the effect of pore size and porosity on separation performance. Additionally, UiO‐66 analogues with engineered lattice defects, namely UiO‐66_F (with missing cluster defects) and UiO‐66_A (with missing linker defects), were also considered to investigate the role of defect engineering in modulating filler–membrane interactions and separation efficiency. Through this controlled and comparative approach, the study aims to elucidate how specific structural features of the MOF fillers (i.e., pore size, defect concentration, and dispersion quality) influence the gas separation properties of the resulting membranes. Particular attention is given to how these features govern polymer–filler interactions, filler integration within the matrix, and ultimately, membrane permeability, and selectivity toward CO_2_.

## 2 Experimental Section

### 2.1 Materials and Reagents

Pebax MH1657 (abbreviated as Pebax; weight ratio PEO:PA, 60:40) was kindly provided by Arkema (France) in colorless pellet form and used without any further treatment. The solution for solvent casting was prepared with ethanol (EtOH; 99%, Merck) and distilled water (d‐H_2_O). Chemicals for the synthesis of UiO‐66‐ and UiO‐67‐type MOFs, including zirconium(IV) chloride (ZrCl_4_), benzene‐1,4‐dicarboxylic acid (or terephthalic acid, H_2_BDC), 4,4‐biphenyldicarboxylic acid (H_2_BPDC), formic acid (FA), acetic acid (AcOH), benzoic acid (BzOH), and N,N‐dimethylformamide (DMF), were supplied by Sigma‐Aldrich and used as received. High‐purity gases (CO_2_, CH_4_, and H_2_ for the permeation tests as well as N_2_, Ar, and He for sorption measurements at 77 K) were purchased from Linde Hellas.

### 2.2 Synthesis of MOF Particles

**Scheme 1:**
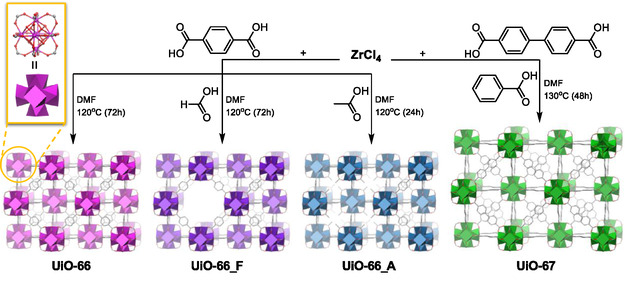
Synthetic procedure of UiO‐66 and its defective analogues UiO‐66_F and UiO‐66_A and its expanded analogue UiO‐67.

All UiO‐type MOFs were synthesized using slightly modified versions of previously reported procedures (UiO‐66,^[^ ^24^ ^]^ UiO‐66_F,^[^ ^24^ ^]^ UiO‐66_A,^[^ ^16^ ^]^ UiO‐67^[^ ^25^ ^]^), as illustrated in **Scheme** **1**. Detailed synthetic procedures are provided in Supporting Information.

### 2.3 Membranes Preparation

Neat and composite Pebax membranes were fabricated using the solution casting method as described in our previous work.^[^ ^13^ ^]^ In particular, 3 wt% Pebax was diluted in an EtOH:H_2_O (70:30) solution and stirred continuously for 8 h at 80 °C under reflux. The polymer solution was placed in plastic petri dish, and the solvent was allowed to evaporate for 48 h at room temperature. The membrane was gently removed from the plastic dish and then dried in a vacuum oven at 40 °C overnight. For the Pebax/UiO‐type MMMs, appropriate amounts of MOF nanoparticles (5, 10, and 20 wt% loading) were initially dispersed into a EtOH:H_2_O (70:30) mixture by continuous stirring at room temperature for 12 h. Afterward, the dispersions were sonicated for 10 min. During the priming step, 20% of the polymer was added, and the solutions were refluxed for 2 h at 80 °C while being continuously stirred. The remaining polymer was added, and the reflux process continued for 6 h at 80 °C. The resulting solutions were sonicated for 10 min and then kept under stirring at room temperature overnight. Finally, the solutions were cast in plastic Petri dishes and placed in a vacuum oven at 40 °C for 8 h after bulk solvent evaporation (48 h at ambient conditions). The thickness of the obtained uniform self‐supported films varied between 50 and 120 μm (for 0 to 20 wt% nanoparticles loading). All synthesized membranes were kept in desiccators until being further tested.

### 2.4 Characterization Methodology

The structural, thermal, and morphological properties of all samples (UiO‐type MOFs and membranes) were studied by X‐ray diffraction (XRD; Rigaku SmartLab, Tokyo, 40 kV, 35 mA, from 2 to 60° with a 0.04 step s^−1^), Fourier transform infrared spectroscopy (FTIR; Thermo Scientific Nicolet 6700 from 400–4000 cm^−1^), thermogravimetric analysis (TGA; SETARAM SETSYS Evolution 18 Analyzer, from 25 to 800 °C, at a heating rate of 10 °C min^−1^ under 16 mL min^−1^ air flow), and scanning electron microscopy (SEM; Jeol JSM 7401 F Field Emission running at 2 kV). MMMs cross‐section samples were prepared by the cryo‐fracturing method using liquid N_2_. Using a sputter coater, an ultrathin Pt layer was then applied to all membrane samples before observation (FISONS Instruments, UK). Backscattered images were acquired with SEM mode at accelerating voltage of 2 kV. Elemental mapping distributions (C, O, Zr) were acquired on carbon‐coated samples by energy dispersive X‐ray spectroscopy (EDS) microanalysis, at acceleration voltage of 10 kV, using an Xplore‐15 SDD detector (Oxford Instruments, Abingdon, UK) with a surface of 15 mm^2^. Modulated differential scanning calorimetry (DSC; 2920 calorimeter, TA Instruments) measurements were performed to evaluate the thermal properties of the prepared membranes, and to investigate the interaction between Pebax and the MOF particles. Two heating cycles were recorded from −35 to 240 °C. All measurements were conducted in nitrogen atmosphere at a heating rate of 5 °C min^−1^ and a temperature modulation of ± 0.80 °C every 60 s. The glass transition temperature (Tg) was determined from separate DSC runs that were performed over the temperature range of −50 to 45 °C at a rate of 10 °C min^−1^ using liquid nitrogen to quench the sample before measurement.

Low‐pressure (up to 1 bar) nitrogen (77 K), argon (87 K), CO_2_ (253, 263, 273, 283, 293 Κ), and CH_4_ (253, 263, 273 Κ) adsorption measurements on the MOF fillers were performed volumetrically on an Autosorb 1‐MP instrument (Quantachrome). Approximately 50 mg of UiO‐type MOFs were used, while isothermal conditions were maintained by means of a Gifford‐McMahon closed cycle refrigeration system (Cryodyne 22, CTI Cryogenics). Ultrahigh‐purity N_2_ (99.999%), Ar (99.999%), CO_2_ (99.995%), CH_4_ (99.999%), and He (99.999%) were used for the adsorption measurements. Prior to analysis, MOF samples were outgassed for 12 h at 220 °C under high vacuum.

Additionally, high‐pressure (up to 20 bar) gas adsorption experiments for the membranes and MOFs were performed at 25 °C on an automated gravimetric system (Intelligent Gravimetric Analyzer IGA‐00, HIDEN ISOCHEMA). Prior to measurement, samples were outgassed under high vacuum for 24 h at 40 °C (membranes) or 120 °C (MOFs).

Single‐gas permeation measurements were performed using a specially designed constant volume/variable pressure system. Circular flat membranes with an effective area of ≈10 cm^2^ were mounted in a stainless‐steel permeation cell and sealed by *O*‐rings. Both sides of the membrane were evacuated for 24 h at 25 °C prior to each measurement. Gas was introduced into the membrane's high‐pressure (feed) side at a pressure of around 2 bar, and the temporal pressure increase at the calibrated, pre‐evacuated permeate volume was monitored. The permeability was calculated from the slope (*dp*/*dt*) of the pressure versus time curve in Barrer (1 Barrer = 10^−10^ cm^3^ (STP) cm cm^−2^ s^−1^ cmHg^−1^) according to Equation (1) and (2).

(1) $$Pe=10^{-10}\frac{J\cdot l}{A\cdot P_{\text{head}}}$$

(2) $$J=\frac{\frac{d_{P}}{d_{t}}\cdot V}{R\cdot T}$$

where *J* is the flow rate (cm^3^ (STP) s^−1^), *l* is the thickness of the membrane (cm), *A* is the membrane's effective area (cm^2^), *P*_head_ is the feed pressure (mbar), *dp*/*dt* is the pressure increase rate on the permeate side (mbar s^−1^), and *V* is the calibrated volume of the permeate side (cm^3^), while *R* and *T* refer to the universal gas constant (0.27821 cm^3^ cmHg cm^−3^ (STP) K^−1^) and temperature (K), respectively. The ideal selectivity (*α*) for a pair of gases (A and B, A being the most permeable) was calculated from single‐gas permeability measurements using Equation (3).

(3) $$a=\frac{Pe_{A}}{Pe_{B}}$$

## 3 Results and Discussion

### 3.1 Characterization of UiO‐Type MOFs and MMMs

To enable a direct comparison of the impact of porosity on membrane performance, we prepared a series of Zr‐based MOFs that share the same structural motif but differ in internal surface area and pore volume as a result of either defect engineering or linker extension. UiO‐66 is a prototypical MOF with *fcu* topology, constructed from [Zr_6_(*μ*_3_‐O)_4_(*μ*_3_‐OH)_4_]^12+^ clusters and terephthalate (BDC) linkers (Scheme 1). This robust coordination results in a microporous framework comprising octahedral (≈11 Å) and tetrahedral (≈8 Å) cavities interconnected through triangular windows (≈7 Å).^[^ ^26^ ^]^ Modulator‐free synthesis yields a highly ordered framework with low concentration of defects. The use of monocarboxylic acid modulators, however, introduces a substantially higher concentration of structural defects, with distinct types depending on the modulator: acetic acid primarily induces missing‐linker defects, while formic acid tends to promote missing‐cluster vacancies, forming nanodomains with reo‐type topology.^[^ ^24^ ^]^ These structural modifications increase internal pore volume and introduce coordinatively unsaturated sites, both of which are known to enhance CO_2_ adsorption capacity and facilitate CO_2_ transport pathways. The isoreticular analogue UiO‐67, incorporating a longer biphenyl dicarboxylate (BPDC) linker, retains the same topology and pore geometry but features significantly larger pores (≈16 and 12 Å for octahedral and tetrahedral cavities, respectively) and a higher specific surface area.^[^ ^26^ ^]^ These structural features are also expected to enhance CO_2_ adsorption and minimize diffusion limitations.

**Figure 1:**
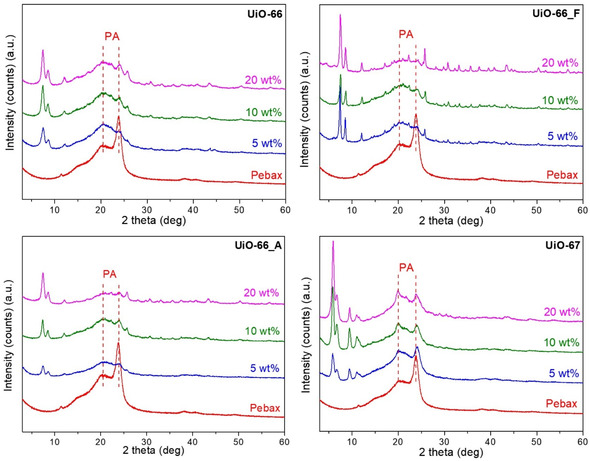
XRD spectra of the neat polymer and the MMMs incorporating Zr–MOFs.

All synthesized MOFs (powder samples) were thoroughly characterized by XRD, SEM, TGA, FTIR, and N_2_ and Ar adsorption–desorption measurements (see detailed analyses in Supporting Information). XRD patterns confirmed the formation of phase‐pure, highly crystalline frameworks for all samples, with the UiO‐66‐based materials displaying characteristic reflections at 7.4° and 8.6° (Figure S3, Supporting Information). As expected, the introduction of defects had a negligible effect on long‐range crystallinity. However, UiO‐66_F exhibited additional low‐angle peaks (at ≈4° and 6°), indicative of partially ordered missing‐cluster regions with reo‐type topology. TGA was employed to quantify the extent of defectivity by estimating the number of missing linkers per Zr_6_ formula unit. The modulator‐free UiO‐66 exhibited low defectivity (≈0.4 missing linkers per Zr_6_ cluster), while UiO‐66_A and UiO‐66_F showed higher defect concentrations of ≈1.0 and 1.5 missing linkers per Zr_6_ unit, respectively, consistent with the introduction of missing‐linker and missing‐cluster defects (Table S1, Supporting Information). UiO‐67 displayed the lowest defect concentration (≈0.1 missing linker per Zr_6_), confirming its near‐ideal framework integrity. N_2_ (77 K) and Ar (87 K) sorption measurements further supported the defect analysis, with BET surface areas and pore volumes increasing across the UiO‐66 series in accordance with defect content. UiO‐66_F showed the highest porosity (1970 m^2^ g^−1^ and 0.74 cm^3^ g^−1^), followed by UiO‐66_A (1450 m^2^ g^−1^ and 0.60 cm^3^ g^−1^) and UiO‐66 (1050 m^2^ g^−1^ and 0.46 cm^3^ g^−1^) (Table S2, Supporting Information). These trends reflect the progressive formation of additional internal void space through defect incorporation. UiO‐67 exhibited the highest overall surface area and pore volume among the materials studied (2410 m^2^ g^−1^ and 0.96 cm^3^ g^−1^), attributed to its intrinsically larger pore size resulting from the longer BPDC linker. SEM imaging revealed that all UiO‐66 samples consist of small nanocrystals with sizes ranging from a few nanometers up to ≈90 nm (Figure S1, Supporting Information). Although their morphology remains partially obscured due to the small crystallite size, no significant morphological differences were observed. In contrast, UiO‐67 forms significantly larger crystallites, ranging from ≈100 to 700 nm, with well‐defined octahedral shapes. Concerning the membranes, **Figure** **1** shows the XRD patterns of all synthesized samples. For the neat Pebax membrane, which is a semicrystalline copolymer consisting of crystalline (PA6) and amorphous (PEO) phases, the characteristic peaks at 20° and 24° assigned to the crystalline PA6 phase of the copolymer, along with a shoulder at ≈15^°^ attributed to the PEO phase, are evident.^[^ ^27^, ^28^, ^29^ ^]^ For the MMMs, it is clear that all Zr–MOFs retained their topology and crystallinity after their incorporation into the polymer matrix (signature peaks: 7.4° and 8.6° for UiO‐66 and its defect‐bearing analogues; 5.7°, 6.7°, and 9.4° for UiO‐67). Although the positions of the Pebax peaks remained unaltered in all MMMs indicating that there were no changes in the d‐spacing of the polymer, their intensity decreased significantly with increasing loadings of UiO‐66 and its analogues, suggesting a reduction in the crystallinity of the impermeable PA6 region of Pebax. Interestingly, while the intensity of the PA6 peaks decreased with UiO‐67 addition, the signal remained unaffected for different loadings of UiO‐67. This suggests that the Pebax‐UiO interface does not change upon increased loading, implying a less efficient dispersion of the large UiO‐67 crystals.

**Figure 2:**
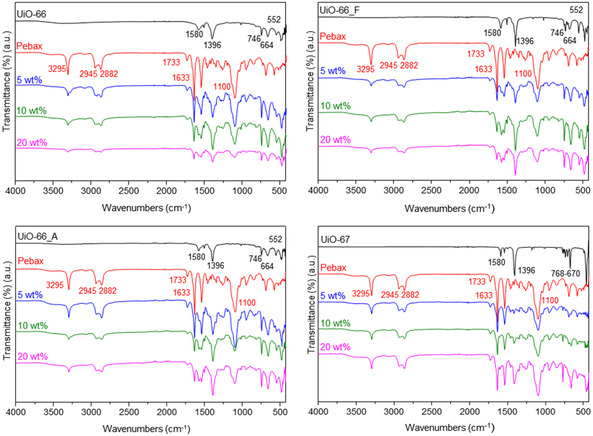
FTIR spectra of the Zr–MOFs, Pebax, and Zr–MOF/Pebax MMMs with 5–20 wt% particle loadings.

FTIR experiments were performed to further investigate the potential bonding between the incorporated UiO‐type MOFs and Pebax chains (**Figure** **2**). The spectra of UiO‐66 and its defective analogues display the characteristic intense peaks at 1580 and 1396 cm^−1^ assigned to the asymmetric and symmetric stretching vibrations of the —COO^−^ group, respectively, in addition to the low energy peaks at 746, 664, and 552 cm^‐1^ associated with vibrations of the zirconium cluster (Zr—O modes).^[^ ^30^, ^31^ ^]^ The UiO‐67 spectrum is very similar to those of the UiO‐66 structures.^[^ ^23^ ^]^ The neat Pebax membrane exhibits multiple distinctive peaks, including those at 3295 cm^−1^ (N—H stretching), 2945 and 2882 cm^−1^ (asymmetric and symmetric C—H vibration), 1733 and 1633 cm^−1^ (C=O stretching of ester and amide), and 1100 cm^−1^ (—C—O—C stretching of ether).^[^ ^18^, ^32^ ^]^ The characteristic peaks of the single components are also present in the spectra of all MMMs. The absence of new peaks in the spectra of the MMMs indicates that UiO nanoparticles are physically wrapped by the polymer chains without strong chemical interactions.

**Figure 3:**
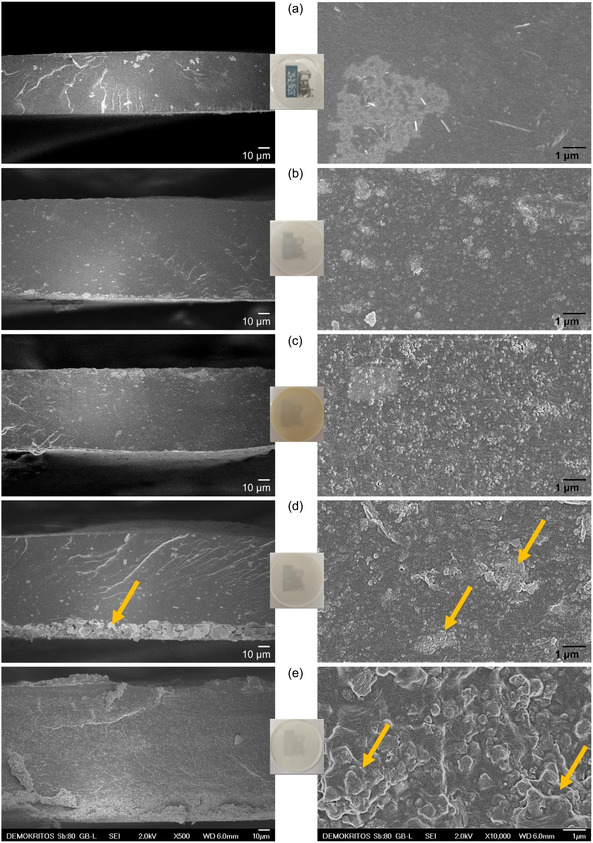
Cross‐sectional SEM images at two magnifications of a) neat Pebax and MMMs containing b) 20 wt% UiO‐66, c) 20 wt% UiO‐66_F, d) 20 wt% UiO‐66_A, and e) 20 wt% UiO‐67. Arrows indicate regions where aggregations of filler particles are observed.

Cross‐sectional SEM images of the neat Pebax membrane (**Figure** **3**a) revealed its rough and nanofibrous surface associated to the two microphases of Pebax. The images of Pebax/20 wt% UiO‐66 (Figure 3b) and Pebax/20 wt% UiO‐66_F (Figure 3c) MMMs show very good dispersion even at such high loadings. The uniform distribution of UiO‐66_F within the Pebax matrix was further supported by EDS elemental mapping and backscattered electron imaging (Figure S9, Supporting Information). In contrast, despite having a similar particle size with UiO‐66 and UiO‐66_F, UiO‐66_A reveal a less ideal dispersion, with noticeable agglomeration on one side of the Pebax/20 wt% UiO‐66_A membrane (Figure 3d). An unsatisfactory dispersion, with extensive filler aggregation, is also observed in the Pebax/20 wt% UiO‐67 membrane (Figure 3e), likely due to the substantially large particle size of UiO‐67.

**Figure 4:**
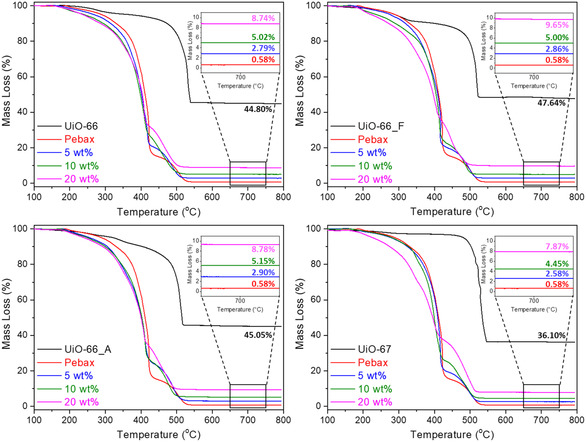
TGA of the Zr–MOFs, Pebax, and Zr‐MOF/Pebax MMMs.

The thermal stability of all synthesized membranes was evaluated by TGA measurements under air flow (**Figure** **4**). Regarding the 3D framework stability and structural integrity of MOFs, all UiO powders exhibited a slight weight loss between 100 and 330 °C, corresponding to the dehydroxylation of the metal cluster, followed by decomposition at ≈500–550 °C with the formation of ZrO_2_.^[^ ^33^, ^34^, ^35^ ^]^ The thermal decomposition of the MMMs began at slightly lower temperatures with increasing MOF content, suggesting minor disruption of the polymer's crystallinity. Importantly, the overall thermal stability remained sufficiently high for gas separation applications, as the decomposition temperatures were still well above typical operating conditions. Furthermore, TGA confirmed that the actual MOF loadings within MMMs fit well with the nominal values (Table S2, Supporting Information).

**Figure 5:**
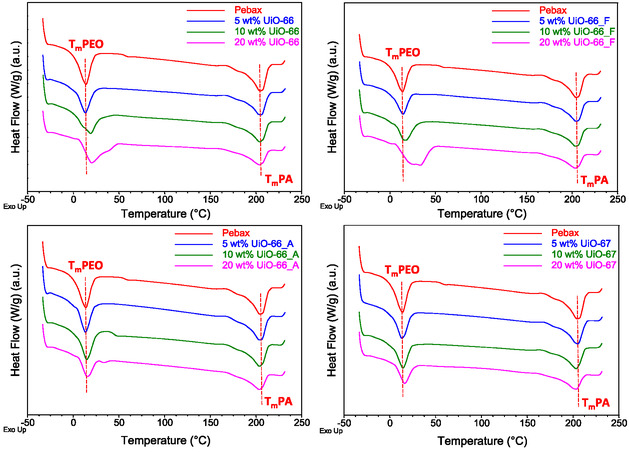
DSC curves of neat Pebax polymer and Zr–MOF/Pebax membranes.

Additional DSC analyses were conducted to assess the effect of incorporating UiO fillers on the glass transition temperature (*T*_g_), melting temperature (*T*_m_), and crystallinity of the composite membranes (**Figure** **5** and **Table** **1**). To ensure accuracy, two heating‐cooling cycles were performed for each sample, and results were obtained from the second cycle to eliminate prior thermal history.

**Table 1:** Thermal properties and crystallinity of neat Pebax and Pebax/UiO systems.

Membrane	Loading	*T*_g_	*T*_m_		Δ*H*_m_		*X*_cryst__._		
	[wt%]	[°C]	[°C]		[J g^−1^]		[%]		
			PEO	PA6	PEO	PA6	PEO	PA6	Total
Pebax	0	−55.4	14.4	204.3	22.8	24.2	22.8	26.3	24.2
UiO‐66	5	−54.7	13.4	203.4	15.4	21.6	16.2	24.7	19.6
	10	−56.3	14.7	203.7	16.2	19.7	18.0	23.8	20.3
	20	−56.3	15.1	203.5	16.9	14.8	21.2	20.1	20.7
UiO‐66_F	5	−57.2	14.2	204.3	14.2	20.7	15.0	23.7	18.5
	10	−57.6	16.6	203.5	15.2	19.9	16.9	24.0	19.8
	20	−56.9	32.1	203.5	23.5	16.3	29.4	22.1	26.5
UiO‐66_A	5	−56.3	13.0	205.5	15.3	21.1	16.1	24.1	19.3
	10	−56.4	18.7	204.0	17.3	19.7	19.3	23.8	21.1
	20	−58.8	19.8	203.6	20.9	15.2	26.2	20.7	24.0
UiO‐67	5	−57.0	13.0	205.1	13.3	21.0	14.0	24.0	18.0
	10	−56.6	14.3	203.3	11.9	18.6	13.2	22.5	16.9
	20	−56.6	16.0	203.2	11.1	14.5	13.9	19.7	16.2

*T*_g_ is a key indicator of polymer chain flexibility or rigidity. For Pebax copolymer, the *T*_g_ point is related with the soft PEO segments, as the *T*_g_ of the hard PA6 segments is not detectable. The pure Pebax membrane exhibited a *T*_g_ of−55.4 °C, which is consistent with literature.^[^ ^36^, ^37^ ^]^ Upon incorporating UiO fillers, a slight reduction in *T*_g_ was observed (Table 1), suggesting that the PEO chains were not significantly rigidified by the MOF particles.^[^ ^37^, ^38^ ^]^

The crystallinity, a factor that can potentially influence the transport properties of the membranes, was also calculated. DSC analysis of pristine Pebax revealed two endothermic peaks at 14.4 and 204.3 °C, corresponding to the *T*_m_ of the PEO and PA6 phases, respectively, in agreement with previous studies.^[^ ^39^ ^]^ The PEO and PA6 melting points were similarly determined for all MMMs and are summarized in Table 1. The apparent latent heat of melting (Δ*H*_m_) of each phase was obtained by integrating the melting peaks in the DSC thermograms. These values were corrected for the MOF loading as well as the PEO and PA content in the blend and were used to calculate the degree of crystallinity (*X*_crystallinity_) according to Equation (4).

(4) $${\text{X}}_{\text{crystallinity}}\text{(\%) = }\frac{\text{Δ}{\text{H}}_{m}}{\text{w}(1‐w_{f})\Delta {\text{H}}_{m}^{0}}\times 100$$

where $\Delta {\text{H}}_{m}^{0}$ is the heat of melting of the 100% crystalline polymer phase ($\Delta {\text{H}}_{m}^{0}$ = 166.4 J g^−1^ for PEO and 230.0 J g^−1^ for PA),^[^ ^40^ ^]^ *w* is the weight fraction of PEO or PA in the sample (0.6 for PEO and 0.4 for PA), and *w*_f_ is the weight fraction of the filler (0.05, 0.10, and 0.20 for the 5, 10, and 20% loading, respectively). The crystallinities of the PEO (*X*_PEO,cryst__._) and PA6 (*X*_PA,cryst__._) phases were then combined to calculate the total crystallinity (*X*_total,cryst__._) of each membrane using Equation (5).^[^ ^41^ ^]^

(5) $$X_{\text{total,cryst}\text{.}}\text{ = 0}\text{.6}\times X_{\text{PEO,cryst}\text{. }}\text{+ 0}\text{.4}\times X_{\text{PA,cryst}\text{.}}$$

The results demonstrate that the incorporation of MOF fillers into Pebax membranes affects the soft (PEO) and hard (PA6) segments differently. The crystallinity of PEO progressively increases with filler loading, remaining though quite close to that of the neat membrane. However, PEO is amorphous at room temperature and thus these variations are not expected to have pronounced effects on membrane performance. Moreover, although a slight increase in PEO's melting point is observed, no significant rigidification occurs (typically associated with strong polymer–filler interactions), indicating that chain mobility is not substantially restricted. In contrast, the crystallinity of the PA segments systematically decreases with increasing filler content, suggesting that the nanoparticles induce local structural perturbations and disrupt chain packing without, however, forming strong chemical bonds, as confirmed by the FTIR analysis. Since the PA phase is the dominant crystalline domain in Pebax, the reduction in its crystallinity enhances the amorphous character of the membrane, increasing its flexibility, which may potentially facilitate the solubility and mobility of penetrants and, ultimately, improve membrane's permeability.^[^ ^42^, ^43^, ^44^ ^]^

### 3.2 Gas Permeation Properties

**Figure 6:**
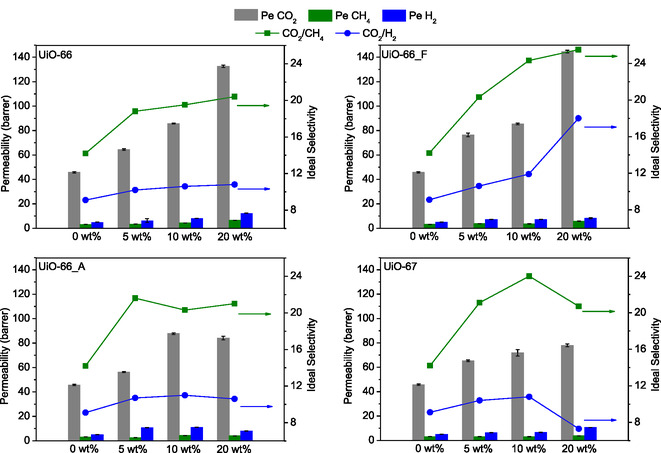
Single‐gas permeability of CO_2_, CH_4_, and H_2_ and ideal selectivity of CO_2_/CH_4_ and CO_2_/H_2_ as a function of MOF loading (wt%) in MMMs. All tests were performed at 25 °C and 2 bar. Error bars represent the standard deviations obtained from 10 experiments.

The gas permeation performance of all synthesized membranes was evaluated by single‐gas permeability tests for CO_2_, CH_4_, and H_2_ at 25 °C under a constant feed pressure of 2 bar (vacuum on the permeate side). Each experiment was repeated 10 times, and the average results are presented in **Figure** **6** and **Table** **2**. The neat polymer membrane exhibited average CO_2_, CH_4_, and H_2_ permeabilities of 45.7, 3.2, and 5.0 Barrer, respectively. The incorporation of the MOF particles into the Pebax matrix resulted in a significant increase in CO_2_ permeability for all membranes: the CO_2_ permeability of the membranes containing 20 wt% UiO‐66 or UiO‐66_F is nearly three times higher than that of the neat Pebax membrane, while the respective values for the membranes with 20 wt% of UiO‐66_A or UiO‐67 are nearly two times higher compared to pure Pebax. Notably, the membrane with 20 wt% UiO‐66_F exhibits a remarkable CO_2_ permeability value of 144.6 Barrer, which is a 216.4% increase over the neat Pebax membrane.

**Table 2:** Single‐gas permeability (*Pe*) at 2 bar and 25 °C, percentage enhancement in CO_2_ permeability (*e*_*Pe*_), and ideal selectivities (*a*).

Membrane	Loading	*P*e CO_2_	*P*e CH_4_	*P*e H_2_	*e*_*Pe*_ a)	*a*	
	[wt%]	[Barrer]	[Barrer]	[Barrer]	CO_2_%	CO_2_/CH_4_	CO_2_/H_2_
**Pebax**	**0**	45.7 ± 0.5	3.2 ± 0.03	5.0 ± 0.04	–	14.3	9.1
**UiO‐66**	**5**	64.4 ± 0.6	3.4 ± 0.05	6.3 ± 1.5	40.9	18.9	10.2
	**10**	85.7 ± 0.4	4.4 ± 0.0	8.1 ± 0.05	87.5	19.5	10.6
	**20**	132.6 ± 1.0	6.5 ± 0.04	12.3 ± 0.09	190.2	20.4	10.8
**UiO‐66_F**	**5**	76.4 ± 1.5	3.8 ± 0.06	7.2 ± 0.03	67.2	20.1	10.6
	**10**	85.4 ± 0.6	3.5 ± 0.06	7.2 ± 0.0	86.9	24.4	11.9
	**20**	144.6 ± 1.1	5.7 ± 0.05	8.1 ± 0.4	216.4	25.4	17.9
**UiO‐66_A**	**5**	56.3 ± 0.4	2.6 ± 0.04	10.7 ± 0.05	23.2	21.7	5.3
	**10**	87.7 ± 0.7	4.3 ± 0.06	11.0 ± 0.05	91.9	20.4	8.0
	**20**	84.2 ± 1.5	4.0 ± 0.04	7.9 ± 0.1	84.1	21.1	10.7
**UiO‐67**	**5**	65.3 ± 0.6	3.1 ± 0.0	6.3 ± 0.09	42.9	21.1	10.4
	**10**	71.9 ± 2.7	3.0 ± 0.03	6.6 ± 0.2	57.3	24.0	10.9
	**20**	78.0 ± 1.2	3.8 ± 0.07	10.7 ± 0.05	70.7	20.5	7.3

a) The enhancement in the single‐gas permeability (*e*_Pe_) due to the incorporation of fillers is defined as ***e***_**Pe**_ = ($\frac{Pe^{\text{MMM}}\;–\;Pe^{P}}{Pe^{P}}$ )×100 (where *Pe* ^MMM^ and *Pe* ^P^ represent the permeability of the MMM and neat Pebax, respectively).

Gas transport in MMMs is mainly governed by the solution‐diffusion mechanism, where the permeability, *Pe*, of a gas species is the product of its solubility, *S*, and diffusivity, *D* (i.e., *Pe = S* × *D*). Solubility is closely related with the gas‐membrane affinity (or else the gas sorption properties of the membrane), while diffusivity is a measure of the mobility of a gas in the membrane. Although H_2_ has the smallest kinetic diameter (2.9 Å) and thus is expected to diffuse significantly faster than CO_2_ (kinetic diameters: CO_2_ = 3.3 Å, CH_4_ = 3.8 Å), its permeability remains lower compared to CO_2_ for all the membranes studied. This is a direct proof that CO_2_ permeability is primarily influenced by its solubility in the membrane rather than its diffusivity.

Poly(ethylene oxide)‐based block copolymers, such as Pebax, are well known for their inherent CO_2_/light gas selectivity due to the strong affinity between CO_2_ and the oxygen atom of the ether groups in the soft PEO segments. The incorporation of nanoporous materials as fillers may further enhance gas permeability and selectivity through different mechanisms, such as 1) disrupting and softening of the matrix, that may lead to enhanced CO_2_ solubility and mobility in the polymer phase around the fillers, 2) enhancing CO_2_ solubility due to preferential CO_2_ sorption in the pores of the filler, and 3) increasing mobility of CO_2_ in the pores of the filler (compared to the polymer phase). For the UiO‐type MOF fillers used in this work, low‐pressure (up to 1 bar) adsorption isotherms (Figure S10, Supporting Information) highlight their strong affinity for CO_2_ (see Figure S12, Supporting Information, for the CO_2_ isosteric heats of adsorption) and therefore their high intrinsic CO_2_ adsorption selectivity, which fully justifies the fact that upon filler loading, the increase in CO_2_ permeability is significantly greater compared to CH_4_ and H_2_, leading thus to enhanced CO_2_/CH_4_ and CO_2_/H_2_ selectivities.

As shown in Table 2, all MMMs (except 20 wt% UiO‐67 for CO_2_/H_2_) outperform the neat Pebax membrane (CO_2_/CH_4_ = 14.3, CO_2_/H_2_ = 9.1). Notably, the membrane containing 20 wt% UiO‐66_F exhibits the highest selectivity among all MMMs for both gas pairs, reaching 25.4 for CO_2_/CH_4_ and 17.9 for CO_2_/H_2_, representing enhancements of 77.6 and 96.7%, respectively. The superior permeability of the MMM containing UiO‐66_F particles is attributed to the presence of missing‐cluster defects in the UiO‐66 framework, which certainly improve CO_2_ adsorption and potentially facilitate CO_2_ permeation pathways. In contrast, the lower permeability observed in the MMMs with UiO‐66_A and in particular with UiO‐67 (i.e., the framework with the most open structure) was attributed to the inadequate filler dispersion within the polymeric matrix, as evidenced by the SEM images.

DSC results show that the incorporation of UiO nanoparticles into the Pebax matrix reduces only slightly the crystallinity of the PA phase of the membranes, presumably by disrupting the hydrogen bonds linking the PA segments. Lower crystallinity is associated with increased polymer chain flexibility, resulting in a “softer” membrane matrix with increased CO_2_ solubility as well as reduced tortuosity and therefore increased gas permeability. However, the small differences in the crystallinity points to a minimal effect of molecular mobility changes upon loading the membranes with the fillers.

**Figure 7:**
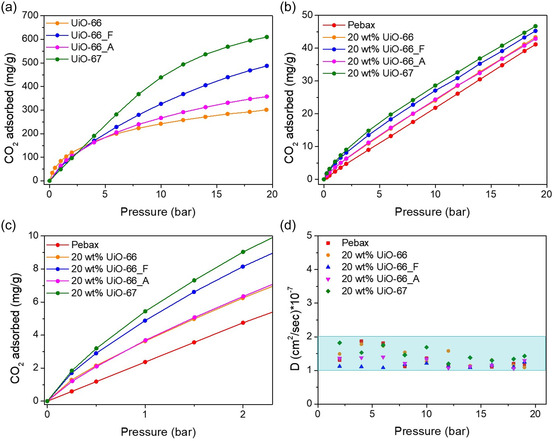
a) CO_2_ isotherms of UiO MOFs measured up to 20 bar at 25 °C, b) CO_2_ isotherms of neat Pebax and Pebax/20 wt% UiO systems measured up to 20 bar at 25 °C, c) corresponding results up to 2.5 bar for clarity, and d) calculated diffusion coefficients of the neat Pebax and 20 wt% UiO MMMs.

To gain further insight into the mechanisms of gas transport and selectivity in MMMs, the solubility and diffusivity of CO_2_ in the membranes (20% loaded with filler) were studied by dynamic adsorption experiments. **Figure** **7** presents the CO_2_ sorption isotherms for UiO‐type MOFs, neat Pebax and Pebax/20 wt% UiO systems measured gravimetrically at 25 °C up to 20 bar, as well as the diffusion coefficients of the membranes, calculated from the sorption kinetic curves recorded during the gravimetric experiments. The UiO MOFs reveal in general type I adsorption isotherms, characteristic of microporous materials, which crossover each other between 2 and 5 bar (Figure 7a). This is because smaller pores lead to enhanced adsorption at low pressures while larger pores provide extra pore volume which fills up at higher pressures. The observed crossover is therefore a direct evidence that the mean pore size of the samples increases in the series UiO‐66 < UiO‐66_A < UiO‐66_F < UiO‐67. Neat Pebax exhibits a linear (Henry‐type) isotherm, typical for dense polymers (Figure 7b,c). The composite membranes display a combination of these two isotherm types (Figure 7b,c), with the addition of UiO nanoparticles enhancing the CO_2_ sorption capacity. However, the increase in sorption capacity is less pronounced than expected, indicating partial pore blocking of the UiO's pore system. This can be due to the wrapping of the Pebax matrix around the crystals or even to poor outgassing of the pore network (e.g., hindered by the polymer). Nevertheless, even though the system is not ideal, the higher amounts of CO_2_ adsorbed by the composites confirm that the addition of UiO MOFs enhances the CO_2_ solubility in the membrane. In contrast, the diffusivity of CO_2_ in the membranes appears unaffected by the presence of the UiO particles (Figure 7d). Thus, the observed increase in CO_2_ permeability and selectivity is attributed solely to the enhanced solubility, driven by the “additional” CO_2_ amount adsorbed by the UiO fillers.

To shed light on the differences in membranes’ performance, it is crucial to consider how the gas transport properties of MMMs are influenced by the morphology of the interface and the transport characteristics of both the continuous polymer matrix and the fillers’ dispersion. Moore and Koros^[^ ^45^ ^]^ and Hashemifard et al.^[^ ^46^ ^]^ have explored nonideal cases in MMM performance that deviate from theoretical predictions of simple models based only on the properties of their pure components. An ideal morphology (Case 0) represents a defect‐free MMM system, where the MMM exhibits improved permeability and selectivity. In contrast, deviations from this ideal state may include polymer rigidification around the filler (Case 1), formation of interfacial voids (Case 2), leakage pathways (Case 3), and partial (Case 4) or severe blockage (Case 5) of filler's pores. Given these premises, the morphology of the interface can be rationalized based on the observed gas permeability and ideal selectivity trends in the UiO‐based MMM systems.

For the 5 wt% UiO‐66 MMM, the CO_2_ permeability increased more than that of CH_4_ and H_2_ (40.9% vs 6.3% and 26.0%), leading to enhanced selectivity compared to pristine Pebax. Higher UiO‐66 loadings exhibited similar trends, albeit less pronounced. The substantial pore blockage observed in CO_2_ sorption measurements, particularly for the 20 wt% UiO‐66 system, suggests that the UiO‐66 MMM behaves, especially at higher loadings, as a combination of Case 0 and 4, i.e., good interfacial contact with partially clogged pores. Nevertheless, a small degree of PEO rigidification around the filler, as evidenced by the broadening of the PEO melting peak (for 10 and 20 wt% loading) in the DSC thermograms (Figure 5), cannot be totally ruled out (Case 1 and 4).

Similarly, Pebax/UiO‐66_F MMMs displayed a linear increase both in terms of CO_2_ permeability and selectivity for filler loadings up to 10 wt%, demonstrating a nearly ideal interfacial morphology (Case 0) with partial pore clogging (Case 4), as indicated by sorption data. At 20 wt% loading, an interfacial change was observed, evidenced by the significant broadening of the PEO melting peak (Figure 5) and manifested as a pronounced increase in CO_2_/H_2_ selectivity.

The UiO‐66_A MMMs exhibited similar performance trends at low filler loadings (e.g., 5 wt%) as UiO‐66 and UiO‐66_F analogues, suggesting an interfacial morphology consistent with Cases 0 and 4. However, at higher loadings, the ideal selectivities do not increase further, while in some cases the permeabilities decrease. This poor performance is likely due to the severe particle agglomeration at one end of the composite membrane, as observed in SEM images (Figure 3d). Such agglomeration significantly hinders gas sorption and diffusion within the UiO crystals.

Finally, the concurrent increase in both CO_2_ permeability and selectivity of 5 wt% UiO‐67 MMM suggests a behavior approaching the ideal interfacial morphology (Case 0), with partial pore clogging (Case 4). However, the increase in CO_2_ permeability becomes less pronounced at higher loadings, while H_2_ permeability increases with increasing filler content. This performance degradation can be attributed to the presence of large filler aggregates observed in SEM cross‐section images (Figure 3e) and the formation of voids at the crystal‐polymer interface (Case 2) leading to a clear leakage of H_2_ (Case 3) at higher loadings that gives a lower CO_2_/H_2_ ideal selectivity compared to the neat Pebax membrane.

### 3.3 Evaluation of MMMs Separation Performance

**Figure 8:**
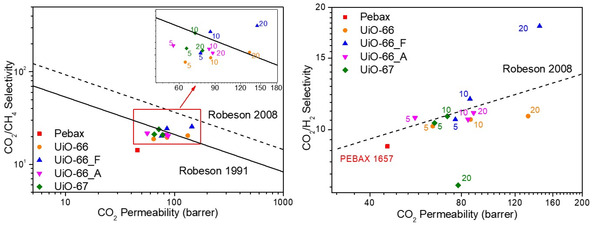
Comparison of MMMs separation performance for CO_2_/CH_4_ (left) and CO_2_/H_2_ (right) on Robeson diagram.

The performance of the Pebax membrane and the MMMs in relation to the 1991 and 2008 Robeson's upper bounds for CO_2_/CH_4_ and CO_2_/H_2_ separations is illustrated in **Figure** **8**. The performance of the neat Pebax membrane lies below the 1991 upper bound for CO_2_/CH_4_ separation. However, the incorporation of UiO nanoparticles into the Pebax matrix enhanced their performance, with the MMMs approaching or slightly exceeding the 1991 upper bound. Among all synthesized MMMs, the Pebax/20 wt% UiO‐66_F membrane demonstrates superior performance, approaching the 2008 upper bound. For CO_2_/H_2_ separation, the studied MMMs achieve performance levels near the 2008 Robeson upper bound, with several of them surpassing it. Notably, the Pebax/20 wt% UiO‐66_F membrane demonstrates again the most significant improvement, exceeding by far the 2008 upper bound. These findings underscore the strong potential of Pebax/UiO MMMs for effective gas separation processes, particularly in CO_2_/CH_4_ and CO_2_/H_2_ applications.

A comparison of the gas separation performance of the best‐performing composite membranes prepared in this work with that of other Pebax 1657‐based MMMs found in the literature is summarized in **Table** **3**. Studies on the gas separation properties of Pebax 1657‐based thin‐film nanocomposite membranes containing UiO‐66 fillers are not included in the table due to differences in testing conditions and membrane configurations.^[^ ^47^, ^48^, ^49^, ^50^ ^]^ As shown in Table 3, the membranes of the present work containing UiO‐66 and UiO‐66_F (20 wt%) exhibit among the highest reported CO_2_ permeabilities at ambient temperature and low‐pressure conditions. Notably, the 20 wt% UiO‐66_F‐based membrane surpasses other reported UiO‐based MMMs, including UiO‐66_NH_2_ (10 wt%)^[^ ^51^ ^]^ and UiO‐66 (10 wt%)^[^ ^51^ ^]^ MMMs. Even at higher UiO‐66 loadings and elevated temperatures or pressures (30 wt%, 65 °C, 10 bar),^[^ ^52^, ^53^ ^]^ the CO_2_ permeability remains only slightly higher than the values obtained in this study at milder conditions. Interestingly, while the 10 wt% KAUST‐7‐based MMM^[^ ^54^ ^]^ exhibits higher absolute permeability compared to our membranes, the percentage enhancement in permeability relative to the neat polymer is significantly lower than in our study.

**Table 3:** Comparison of Pebax 1657‐based MMMs containing different MOFs.

Filler		Condition		Performance				Reference
MOF type	Loading	T	P	*Pe* CO_2_	*e*_*Pe*_	Ideal selectivity [*a*]		
	[wt%]	[°C]	[bar]	[Barrer]	CO_2_%	CO_2_/CH_4_	CO_2_/H_2_	
UiO‐66	20	25	2	133	190	20.4	10.8	This work
UiO‐66_F	20	25	2	145	216	25.4	17.9	This work
UiO‐66	10	20	3	98	16	22.1	–	[51]
UiO‐66‐NH_2_	10	20	3	118	36	30.5	–	[51]
UiO‐66	30	65	10	156	90	17.0	–	[52]
UiO‐66@IL	30	65	10	124	51	33.4	–	[52]
UiO‐66‐NH_2_@IL	30	65	10	135	65	34.4	–	[52]
UiO‐66	30	20	10	156	90	17		[53]
PANI@UiO‐66	30	25	10	119	45	27	–	[53]
Cu‐BTC	3	25	1	119	342	16.2	–	[55]
sub‐NH_2_‐Cu‐BTC	3	25	1	109	303	29.1	–	[55]
MIL‐53(Al)	10	35	10	129	137	23.3	12.1	[56]
NH_2_‐MIL‐53(Al)	10	35	10	149	174	20.5	10.6	[56]
KAUST‐7	10	35	2	163	79	–	10.1	[54]
KAUST‐7	15	35	2	115	26	–	10.6	[54]

Regarding ideal CO_2_/CH_4_ selectivity, the UiO‐66_F MMM ranks among the highest for UiO‐based MMMs, outperforming UiO‐66 and exceeding values reported for other Pebax‐based MMM, such as Cu‐BTC^[^ ^55^ ^]^ and NH_2_‐MIL‐53(Al).^[^ ^56^ ^]^ However, its selectivity remains lower than that of UiO‐66‐NH_2_‐,^[^ ^51^ ^]^ UiO‐66@IL‐,^[^ ^52^ ^]^ UiO‐66‐NH_2_@IL‐,^[^ ^52^ ^]^ PANI@UiO‐66‐,^[^ ^53^ ^]^ and sub‐NH_2_‐Cu‐BTC^[^ ^55^ ^]^‐based MMM, all of which achieve stronger selectivity at the expense of permeability, underscoring the favorable balance between permeability and selectivity attained in this work. For CO_2_/H_2_ selectivity, the UiO‐66_F MMM demonstrates significantly higher selectivity than UiO‐66‐ and other MOF‐containing Pebax‐based MMMs, such as NH_2_‐MIL‐53(Al)^[^ ^56^ ^]^ and KAUST‐7.^[^ ^54^ ^]^ These results highlight the exceptional balance between permeability and selectivity achieved with UiO‐66_F, apparently due to the improved filler dispersion and the presence of missing‐cluster defects, which enhance CO_2_ transport by providing additional adsorption and diffusion pathways.

## 4 Conclusions

This work investigated the development of Pebax MH1657‐based MMMs incorporating a series of UiO‐type Zr‐based MOFs, including UiO‐66, UiO‐67, and two defect‐engineered UiO‐66 analogues (UiO‐66_A and UiO‐66_F), for enhanced CO_2_ separation performance. The main aim was to systematically assess how structural features of the fillers, such as pore size, porosity, and defect type and as well as the polymer‐filler compatibility, influence gas transport behavior in dense MMMs. Membranes were fabricated via solution casting with varying filler loadings (5–20 wt%), and their structural, morphological, and thermal properties were thoroughly studied. The impact of incorporating UiO nanoparticles into the polymer matrix on gas separation properties was systematically assessed through single‐gas permeation experiments.

DSC revealed that increasing MOF loading disrupted hydrogen bonding between Pebax's PA segments, reducing PA6 crystallinity and leading to higher CO_2_, CH_4_, and H_2_ permeabilities. This decrease in crystallinity is associated with enhanced polymer chain flexibility, which facilitates gas transport by lowering diffusion resistance. Additionally, sorption experiments indicated partial blockage of UiO pores, suggesting that while MOF adsorption sites contribute to permeability, their accessibility may be somewhat restricted. Nevertheless, gas permeation measurements revealed that incorporation of UiO particles into the Pebax matrix led to a steady enhancement in CO_2_ permeability across all MMMs. Among these, the 20 wt% UiO66‐F‐ (i.e., the MOF containing missing‐cluster defects) loaded MMM exhibited the best combination of CO_2_ permeability (146 Barrer) and ideal CO_2_/CH_4_ (25.4) and CO_2_/H_2_ (17.9) selectivities. Compared to other Pebax‐based MMMs reported in the literature, UiO‐66_F demonstrates a favorable permeability‐selectivity balance, achieving higher CO_2_ permeability while maintaining competitive selectivity under mild conditions, making it particularly attractive for practical gas separation applications. This superior performance is attributed to the combination of increase internal free volume (due to the missing‐cluster defects) and, more importantly, to its uniform dispersion within the matrix, which enhances CO_2_ sorption capacity and transport pathways, resulting in improved CO_2_ selectivity over CH_4_ and H_2_.

The results underscore that beyond intrinsic MOF porosity, the effectiveness of MMMs is closely tied to how well fillers are integrated into the polymer matrix. The combination of defect engineering and good dispersion was found to be especially important for maximizing the gas separation performance of MOF‐based MMMs, providing a valuable framework for the design of future composite membranes.

## Conflict of Interest

The authors declare no conflict of interest.

## Supporting information

Supplementary Material

## Data Availability

The data that support the findings of this study are available from the corresponding author upon reasonable request.
