# Supplementary material for: UiO‐Based Mixed Matrix Membranes for Efficient CO2 Separations
Source: Chempluschem. 2025 May 29;90(7):e202500151. doi: 10.1002/cplu.202500151 (PMC12261053; doi:10.1002/cplu.202500151)
Supplement: Supplementary file 1 — Supplementary Material [file CPLU-90-e202500151-s001.pdf]

UiO-based Mixed Matrix Membranes for Efficient CO_2 Separations

Lamprini G. Boutsika,[a]Christos Tampaxis,[a]KyriakiPapadokostaki,[a]Merope Sanopoulou,[a]Georgia Charalambopoulou,[b]Ioannis Bratsos,*[a]Theodore Steriotis[a]

[a] Institute of Nanoscience and Nanotechnology, National Centre for Scientific Research "Demokritos", 15341 Ag. ParaskeviAttikis, Greece

[b] Institute of Nuclear & Radiological Sciences & Technology, Energy & Safety, National Centre for Scientific Research "Demokritos", 15341 Ag. ParaskeviAttikis, Greece

* Corresponding author: i.bratsos@inn.demokritos.gr

SUPPORTING INFORMATION

Table of Contents

S1. Synthesis of UiO-type MOF particles S2
S2. Physicochemical Properties of MOFs S3
S2.1 Scanning Electron Microscopy (SEM) S3
S2.2 Thermogravimetric Analysis (TGA) S3
S2.3 X-Ray Diffraction (XRD) S7
S2.4 Nitrogen/Argon porosimetry S8
S3. Physicochemical Properties of MMMs S13
S3.1 Scanning Electron Microscopy (SEM) S13
S3.2 Thermogravimetric Analysis (TGA) S14
S4. Gas adsorption measurements - isosteric heat of adsorption S15
S5. References S19

S1. Synthesis of UiO-type MOF particles

UiO-66: In a 500 mL screw-top glass bottle, 1.7240 g (7.4 mmol) $ZrCl_4$, 0.4 mL d-H_2O and 1.2297 g (7.4 mmol) H_2BDC were added in 200 mL DMF followed by vigorous stirring and heating (ca.70°C) to ensure complete dissolution. The solution mixture was then placed into an oven and heated to 120°C for 72 h. The resulting gel-like product was collected by centrifugation (9000 rpm; 15 min) and washed with DMF (×3) and acetone (×3). The white product was dried overnight at 200°C. Yield = 1.8652 g (90.1% based on Zr(IV) and the formula $[Zr_6O_4(OH)_4(BDC)_6]$).

UiO-66_F: In a 500 mL screw-top glass bottle, 1.7240 g (7.4 mmol) $ZrCl_4$, 0.4 mL d-H_2O, 27.9 mL FA (740.2 mmol; 100 equiv.) and 1.2297 g (7.4 mmol) H_2BDC were added in 180 mL of DMF. The solution mixture was stirred at 70°C for 20 min, and then placed into an oven and heated to 120°C for 72 h. The resulting microcrystalline white powder was collected by centrifugation (9000 rpm; 30 min), washed with DMF (×3) and acetone (×3), and dried overnight at 200°C. Yield = 1.7325 g (84.4% based on Zr(IV) and the formula $[Zr_6O_4(OH)_4(BDC)_6]$, i.e. without taking into account any defects).

UiO-66_A: In a 500 mL screw-top glass bottle, 1.6900 g (7.3 mmol) $ZrCl_4$, 0.4 mL d-H_2O, 21.0 mL AcOH (367.5 mmol; 50.3 equiv.), and 1.2100 g (7.3 mmol) H_2BDC were added in 200 mL DMF. The solution was vigorously stirred at 70°C for 20 min and then placed into an oven and heated to 120°C for 24 h. The resulting microcrystalline white powder was recovered by centrifugation (9000 rpm; 30 min), washed with DMF (×3) and acetone (×3), and dried overnight at 200°C. Yield = 1.2860 g (63.5% based on Zr(IV) and the formula $[Zr_6O_4(OH)_4(BDC)_6]$, i.e. without taking into account any defects).

UiO-67: In a 100 mL screw-top glass bottle, 0.8622 g (3.7 mmol) $ZrCl_4$ and 1.3560 g (11.1 mmol; 3 equiv.) BzOH were dissolved in 14.3 ml DMF containing 0.2 ml H_2O after mild heating (70°C) and stirring for 30 min. Then, 0.8962 g (3.7 mmol) H_2BPDC was added, and once all reagents dissolved, the solution mixture was heated to 130°C for 48 h under stirring. The formed microcrystalline white powder was collected by centrifugation (9000 rpm; 1 min), washed with DMF (×3) and acetone (×3), and dried overnight in an oven set to 200°C. Yield = 1.2007 g (91.8% based on Zr(IV) and the formula $[Zr_6O_4(OH)_4(BPDC)_6]$).

S2. Physicochemical Properties of MOFs

S2.1 Scanning Electron Microscopy (SEM)

The SEM images of the synthesized MOFs (Fig. S1) show that UiO-66, UiO-66_A, and UiO-66_F are composed of small nanocrystals with sizes ranging from a few nanometers up to ca. 90 nm. While these materials appear to adopt an octahedral morphology, the small crystallite dimensions make it difficult to resolve their shape clearly at this scale. However, no significant morphological variation among UiO-66s is observed. On the other hand, UiO-67 exhibits significantly larger crystallites, with broad size distribution ranging from ca. 100 to 700 nm, with well-defined octahedral geometry.

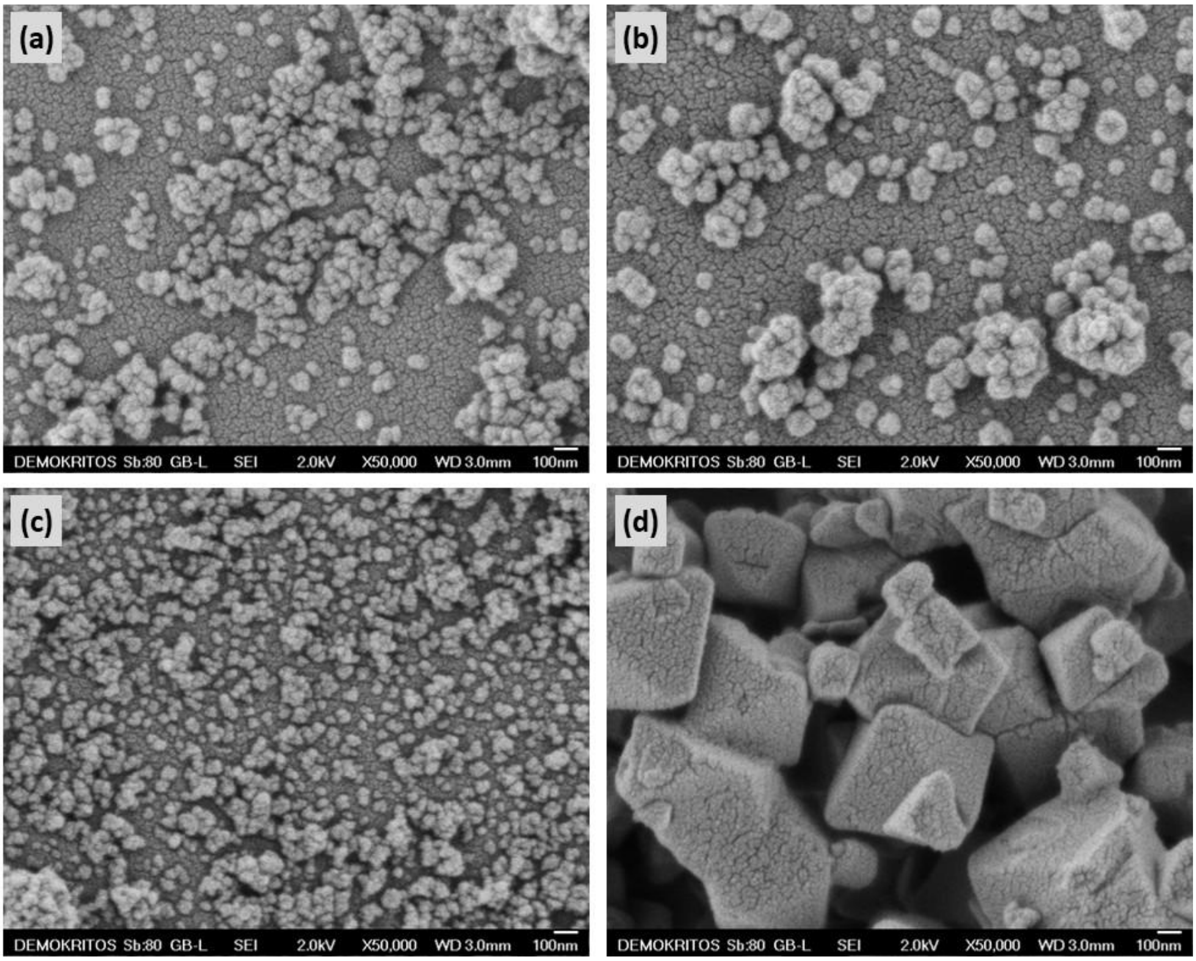

Figure S1. SEM images of (a) UiO-66, (b) UiO-66_F, (c) UiO-66_A, and (d) UiO-67. (The roughness observed on the surface of the crystallites and on the wafer is an artifact resulting from the Pt coating)

S2.2 Thermogravimetric Analysis (TGA)

The TGA results for the synthesized UiO samples are shown in Figure S2. In order to compare and analyze the data quantitatively, the curves were normalized to end weight of

100%. Three well-resolved weight loss steps can be observed: i) a weight loss up to ca. 100°C due to the removal of solvent guest molecules, ii) a gradual loss between ca. 200 and 390°C, attributed to the concurrent release of residual DMF, structural water (i.e. dehydroxylation), and modulator molecules, and iii) a significant weight loss between ca. 390 and 550°C, associated with the framework decomposition via combustion of the organic linkers and the formation of ZrO_2 as the final residue.

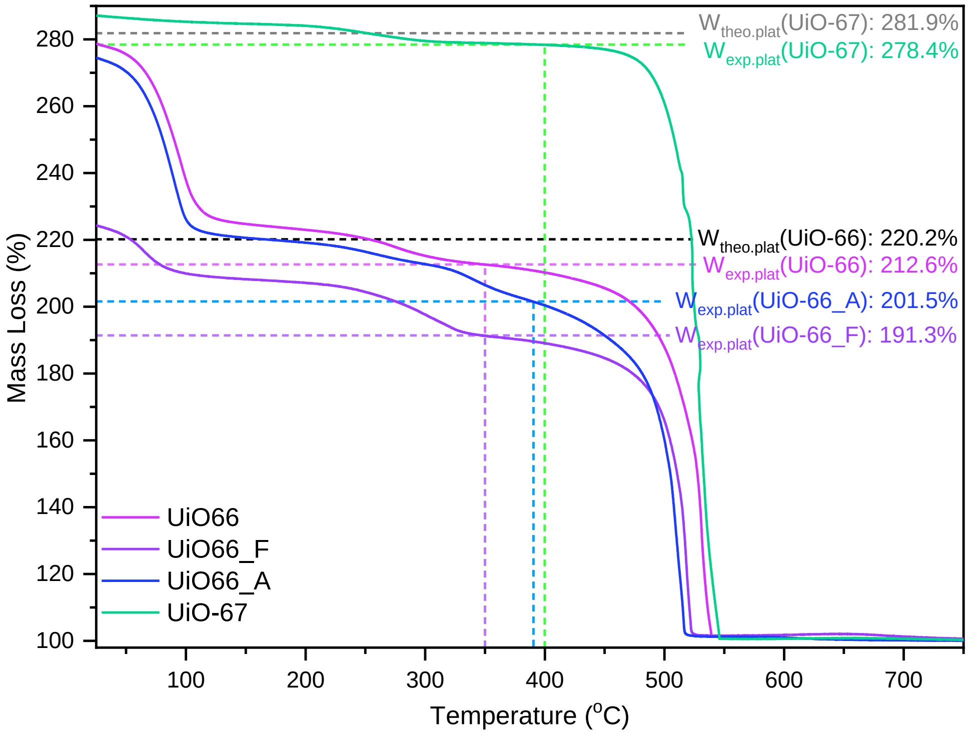

Figure S2. TG analysis of the synthesized MOFs, normalized to end weight of 100%. The vertical dashed lines indicate the temperature (T_{plat}) at which the plateau is reached, i.e where the material has lost everything (solvents, hydroxyl groups, and modulators) but the linkers (magenta and purple: T_{plat} = 350°C for UiO-66 and UiO-66_F, respectively; blue: T_{plat} = 390°C for UiO-66_A; green: T_{plat} = 400°C for UiO-67). The horizontal dashed lines pinpoint the relevant TGA plateaus ($W_{exp.plat}$).

From Fig. S2, it is evident that the magnitude of the decomposition weight loss in all samples is significantly lower than that theoretically expected (compare horizontal dashed lines marked $W_{theo.plat}$ *vs.* $W_{exp.plat}$). In other words, all synthesized UiO materials are lighter than the ideal dehydroxylated UiO-66 indicating that they are linker deficient, as it is well-established that the magnitude of the decomposition weight loss, when normalized as above, is inversely correlated with the defectivity of the material.[24] It should be noted here, that TGA alone cannot distinguish between missing-linker and missing-cluster defects, as both result in a reduced number of linkers. Among the UiO-66 samples, the magnitude of the decomposition weight loss (i.e., combustion of the organic linkers) decreases in the order UiO-66 > UiO-66_A > UiO-

66_F. This trend indicates a progressive reduction in organic content, which directly corresponds to an increasing degree of defectivity in the same order. That is, the more defective the framework, the fewer linkers are present, and thus the smaller the mass loss observed during decomposition.

To quantitatively compare the three UiO-66 analogues, the number of linker deficiencies per Zr_6 cluster was calculated following the method proposed by Shearer *et al*. [24] Taking into account the assumption that the residue in the TGA experiment is pure ZrO_2, and that the reaction of the complete combustion of ideal (defect-free), dehydroxylated UiO-66, $Zr_6O_6(C_8H_4O_4)_6$ is:

$$Zr_6O_6(C_8H_4O_4)_6\,(s) + 45\ O_2\,(g) \rightarrow 6\ ZrO_2\,(s) + 48\ CO_2\,(g) + 12\ H_2O\,(g)$$

the theoretical TGA plateau weight ($W_{Theo.Plat}$) can be determined from the Eq. S1:

$$W_{theo.plat} = \frac{M_{UiO-66-dehydr}}{M_{6xZrO2}} \times W_{end} \qquad (S1)$$

where $M_{UiO\text{-}66\text{-}dehydr}$ is the molar mass of ideal (defect-free) dehydroxylated UiO-66 (M_W = 1628.03 g/mol), M_{6xZrO2} is the molar mass of 6 moles of zirconium oxide (6×123.22=739.34 g/mol), and W_{end} is the normalized end weight (i.e. the weight at 800°C) of the TGA run (= 100%). That is $W_{theo.plat}$(UiO-66) = 220.2% (black horizontal dashed-line in Fig. S2). To quantify the number of linker deficiencies per Zr_6 formula unit in the synthesized UiO-66 samples, the general formula for a defective UiO-66 sample in which everything (solvents, hydroxyl groups, and modulators) but the linker has been lost (i.e. $Zr_6O_{6+x}(BDC)_{6-x}$; x is the number of linker deficiencies per Zr_6 formula unit), as well as the weight contribution per linker $Wt.PL_{Theo}$ (calculated from the equation: $Wt.PL_{theo} = (W_{theo.plat} - W_{end})/NL_{Ideal}$ = (220.2-100)/6 = 20.03%) are considered. Now, the actual (experimental) average number of linkers per defective Zr_6 formula unit (NL_{exp}) can be determined by the Eq. S2:

$$NL_{exp} = (6\text{-}x) = \frac{W_{exp.plat} - W_{end}}{Wt.PL_{theo}} \qquad (S2)$$

where $W_{exp.plat}$ is the experimental weight of the TGA plateau at the temperature (T_{plat}) where non-linker components (solvents, hydroxyl groups, and modulators) are lost (T_{plat} = 350°C for UiO-66 and UiO-66_F; 390°C for UiO-66_A). From Fig. S2 can be seen that the $W_{exp.plat}$ for the modulator-free UiO-66 is 212.6% (magenta horizontal dashed line), for the UiO-66_F is 191.3% (purple horizontal dashed line), whereas for UiO-66_A is 201.5% (blue horizontal dashed line). Thus,

- UiO-66 (modulator-free): NL_{exp} = (212.6–100)/20.03) = 5.62

- UiO-66_F: NL_{exp} = (191.3–100)/20.03) = 4.56
- UiO-66_A: NL_{exp} = (201.5–100)/20.03) = 5.07

Solving equation S2 for x (i.e. x = 6–NL_{exp}), the number of linker deficiencies per Zr_6 formula unit for each MOF can be calculated:

- UiO-66 (modulator-free): x = 6 – 5.62 = 0.38
- UiO-66_F: x = 6 – 4.56 = 1.44
- UiO-66_A: x = 6 – 5.07 = 0.93

If the x values are inserted into the general formula $Zr_6O_{6+x}(BDC)_{6-x}$, the composition of the samples at $W_{exp.plat}$, can be estimated (Table 2). As expected, the modulator-free UiO-66 exhibits the lowest level of defectivity with only ca. 0.4 linker missing per Zr_6 cluster. On the other hand, the modulated UiO-66's are highly defective: UiO-66_A is missing ca. 1 linker for each Zr_6 cluster, whereas UiO-66_F is the most linker deficient, and thus the most defective (ca. 1.5 missing linkers per Zr_6 cluster), due to the presence of missing cluster defects (see section S2.3 X-Ray Diffraction (XRD)).

Following the same method, the deficiency of UiO-67 was estimated, given that the complete combustion of ideal (defect-free), dehydroxylated UiO-67, $Zr_6O_6(C_{14}H_8O_4)_6$ is:

$$Zr_6O_6(C_{14}H_8O_4)_6 (s) + 87\ O_2 (g) \rightarrow 6\ ZrO_2 (s) + 84\ CO_2 (g) + 24\ H_2O (g)$$

as well as that the molar mass of ideal (defect-free) dehydroxylated UiO-67 is 2084.57 g/mol, the $W_{theo.plat}$(UiO-67) is 282.0% (gray horizontal dashed-line in Fig. S2), the Wt.PL_{theo} (= ($W_{theo.plat}$–W_{end})/NL_{Ideal} = (282.0–100)/6) = 30.33%, and the $W_{exp.plat}$ (278.4%; green horizontal dashed-line in Fig. S2) was determined at T_{plat} = 400°C, where the material has lost everything (solvents, hydroxyl groups, and benzoic acid modulator) except the linkers.

According to Eq. S2:

NL_{exp} = (278.4–100)/30.33) = 5.88 and x = 6 – 5.88 = 0.12

Inserting the x value into the general formula $Zr_6O_{6+x}(BPDC)_{6-x}$, the composition of the sample at $W_{exp.plat}$ is obtained (Table S1). These results indicate that UiO-67 is only marginally defective.

Table S1. Quantititative TGA analysis of dehydoxylated UiO-66 analogues..

Sample	T_{plat} / °C	$W_{exp.plat}$ / %	x	NL_{exp} (6-x)	Composition at $W_{exp.plat}$
UiO-66	350	212.6	0.38	5.62	$Zr_6O_{6.38}(BDC)_{5.62}$
UiO-66_F	350	191.3	1.44	4.56	$Zr_6O_{7.44}(BDC)_{4.56}$

UiO-66_A	390	201.5	0.93	5.07	$Zr_6O_{6.93}(BDC)_{5.07}$
UiO-67	400	268.7	0.12	5.88	$Zr_6O_{6.12}(BPDC)_{5.88}$

S2.3 X-Ray Diffraction (XRD)

XRD analysis was carried out to investigate the crystallinity and the phase purity of the synthesized MOFs. The XRD pattern of UiO-66 and its derivatives display signature peaks at 7.4° and 8.6°, corresponding to the (111) and (200) planes, respectively, which are consistent with the simulated pattern for UiO-66 crystal structure (Fig. S3). As expected, the introduction of defects, particularly missing-linker types, does not significantly alter the XRD patterns, as such defects are typically disordered and do not affect the long-range periodicity of the framework.[57] However, the XRD pattern of UiO-66_F exhibits two additional weak and broad peaks centered at ca. 4° and 6°, which are absent in the other UiO-66 samples. These low-angle reflections are associated with the presence of *reo*-type nanodomains, indicative of partially ordered missing-cluster defects within the UiO framework.[57] The XRD pattern of UiO-67 (Fig. S4) showed three sharp peaks at 5.7° (111), 6.7° (200) and 9.4° (220) which are in good agreement with the simulated pattern for UiO-67 crystal structure.[58]

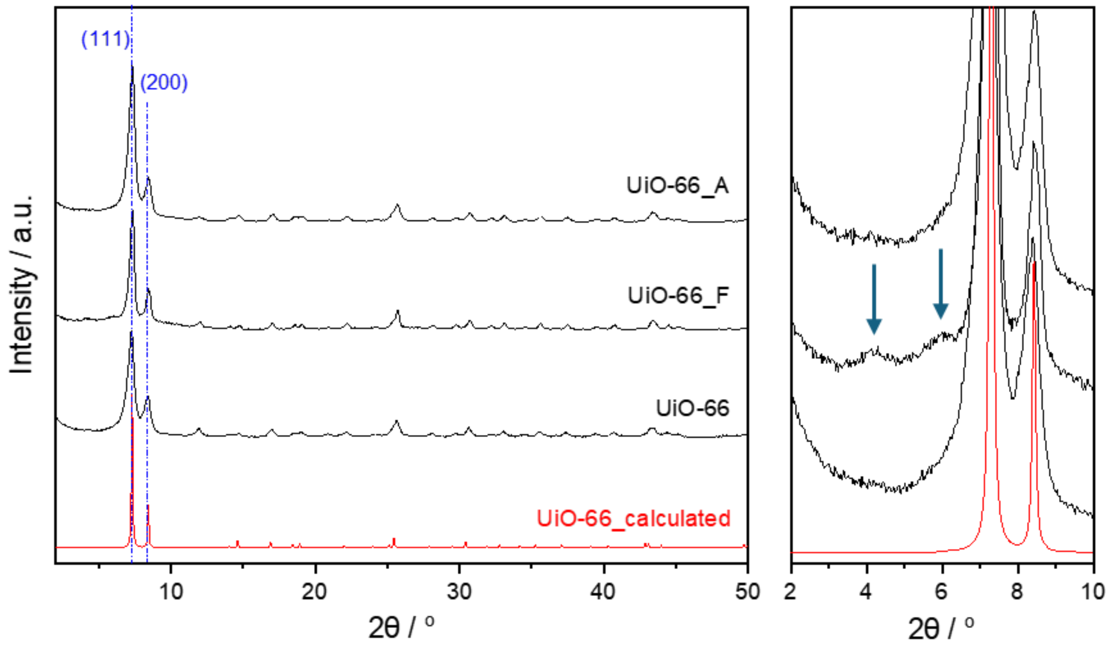

Figure S3. Left: The XRD patterns of the as-synthesized UiO-66, UiO-66_F and UiO-66_A in comparison to the calculated one. Right: The same patterns focused on the range of 2-10° and magnified. The arrows highlight the broad and weak peaks at ca. 4° and 6°, that appear in the diffractogram of UiO-66_F, indicative of missing-cluster defects.

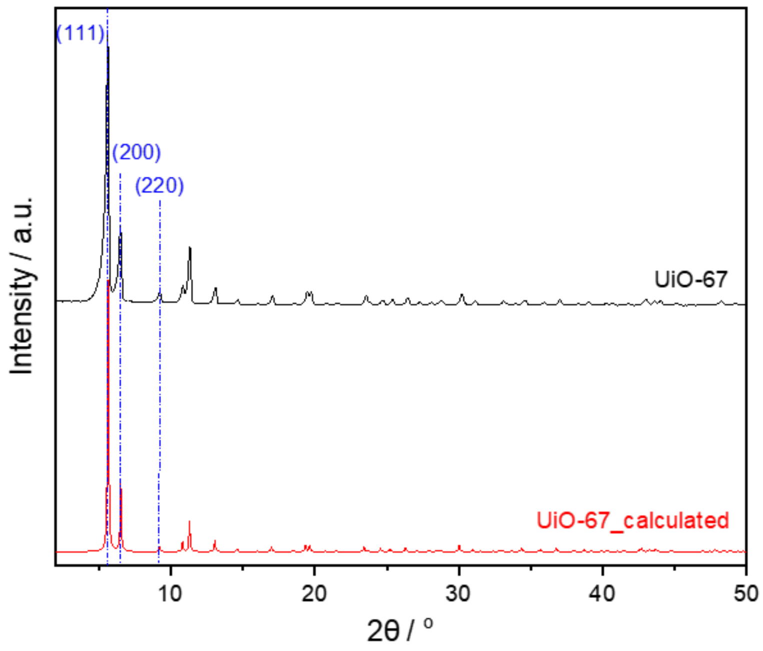

Figure S4. The XRD pattern of UiO-67 in comparison to the calculated one.

S2.4 Nitrogen/Argon porosimetry

The nitrogen (77K) and argon (87K) adsorption-desorption isotherms of all samples exhibit the general characteristics of type-I isotherms (i.e., pore filling that leads to steep uptake at low relative pressures followed by an adsorption plateau), typical of microporous materials (Fig. S5 – S8). Notably, the isotherms of UiO-66_A (Fig. S7) and especially UiO-66_F (Fig. S6) tend to be of type-I(b), which refers to materials with pore size distributions that include wider micropores and possibly very narrow mesopores. This is consistent with the presence of structural defects deliberately introduced during synthesis. In particular, UiO-66_F, prepared using excess formic acid as a modulator to promote cluster vacancies, exhibits a significant increase in both BET surface area and pore volume compared to modulator-free UiO-66 (Table S2), which, while not perfectly defect-free, is known to possess a highly ordered structure with minimal intrinsic defectivity relative to modulated analogues.[24] This enhancement is more pronounced than that observed for UiO-66_A, which contains mainly missing-linker defects. The greater increase in pore volume in UiO-66_F reflects the formation of larger internal voids within the framework, a hallmark of missing-cluster defects that involve the removal of entire Zr_6 nodes and generate secondary pore space. Furthermore, the isotherms of UiO-66 (Fig. S5) and UiO-66_A (Fig. S7) show capillary condensation at high relative pressures, along with

desorption hysteresis (type-IV isotherm shape) indicating the presence of large mesopores, [59] attributed to nanosized voids between aggregated UiO crystallites (inter-crystalline porosity).

The isotherms of UiO-67 (Fig. S8), the isoreticular analogue of UiO-66 incorporating the longer BPDC linker, exhibit the highest overall uptake, BET area and pore volume among all studied here MOFs, consistent with its larger pore size due to framework expansion.

The BET areas and pore volumes of all UiO materials, calculated from both nitrogen and argon isotherms, are summarized in Table S2. The data confirm the relative degree and nature of defectivity in the UiO-66 series, where increasing porosity reflects greater structural openness associated with the type and extent of defects introduced. Together with UiO-67, which exhibits high porosity due to its inherently expanded framework rather than defect formation, the data collectively underscore that both crystallographic design and defect modulation were effectively used to tailor the textural properties of MOFs studied.

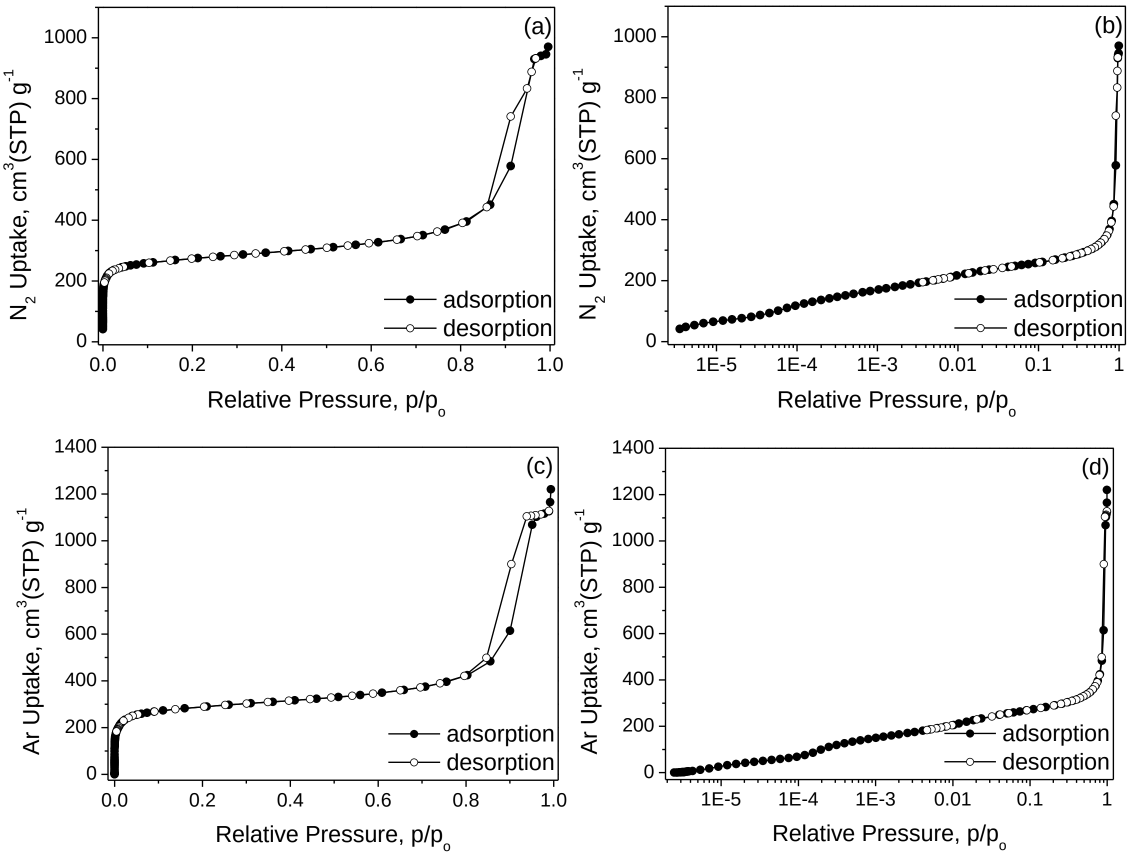

Figure S5. Nitrogen (a, b) and argon (c, d) adsorption/desorption isotherms of UiO-66 recorded at 77 and 87K, respectively, in linear (a, c) and semi-log (b, d) scale.

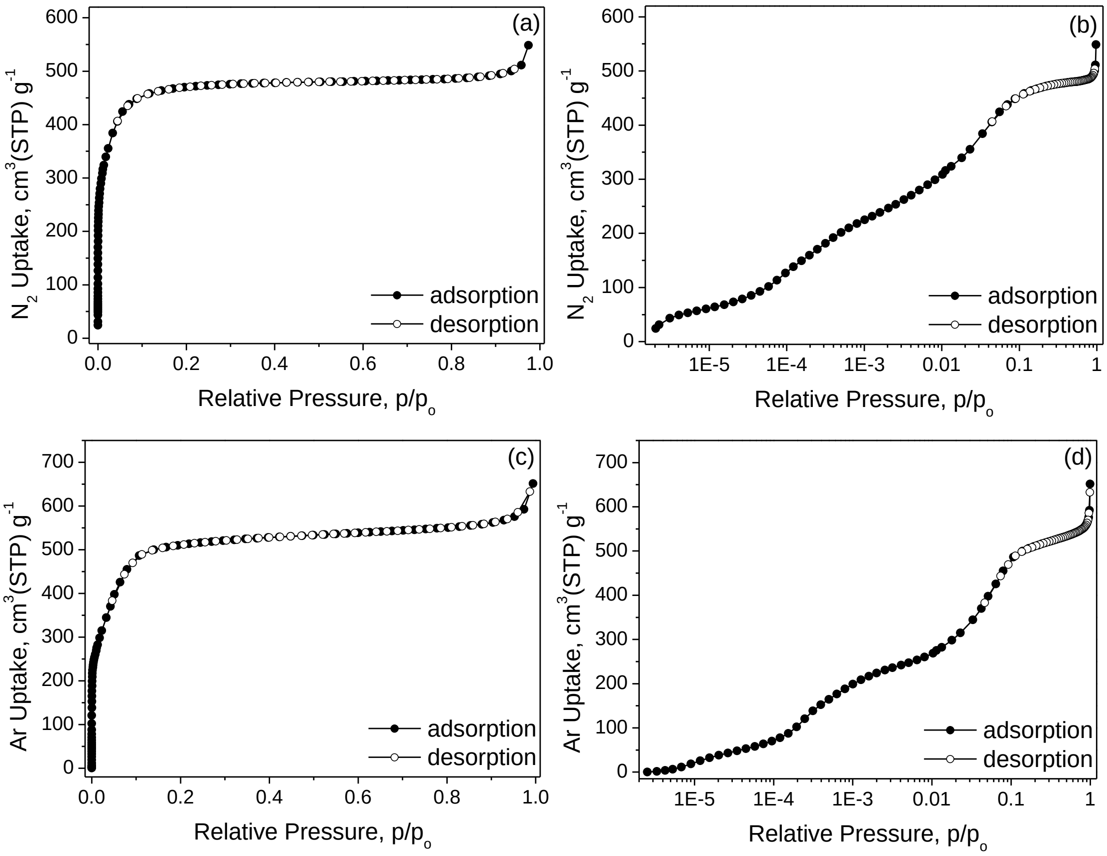

Figure S6. Nitrogen (a, b) and argon (c, d) adsorption/desorption isotherms of UiO-66_F recorded at 77 and 87K, respectively, in linear (a, c) and semi-log (b, d) scale.

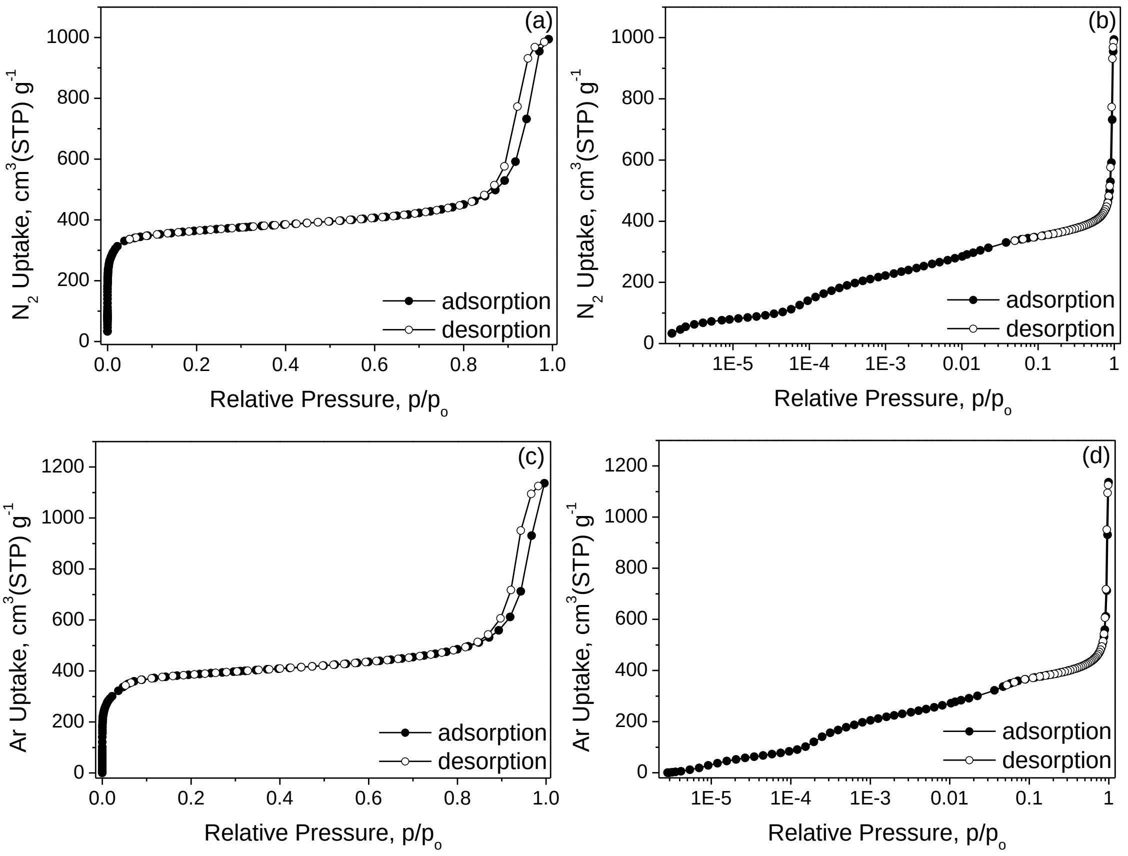

Figure S7. Nitrogen (a, b) and argon (c, d) adsorption/desorption isotherms of UiO-66_A recorded at 77 and 87K, respectively, in linear (a, c) and semi-log (b, d) scale.

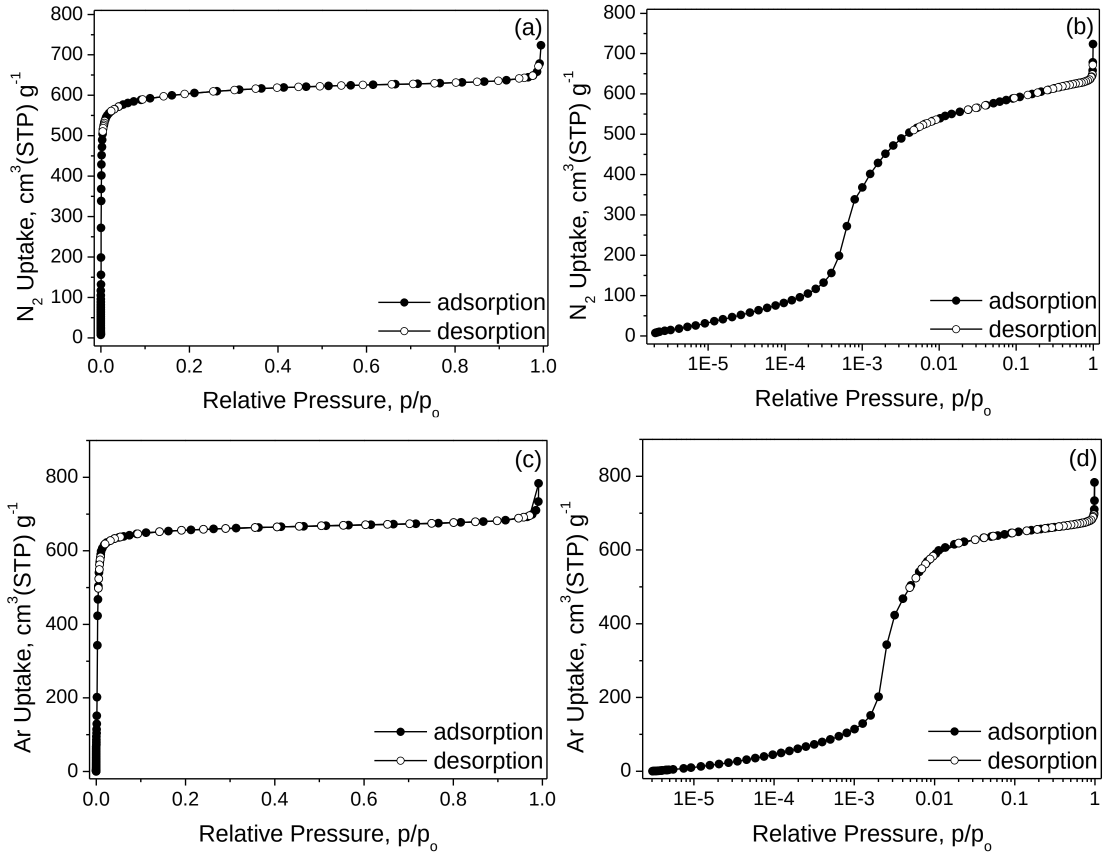

Figure S8. Nitrogen (a, b) and argon (c, d) adsorption/desorption isotherms of UiO-67 recorded at 77 and 87K, respectively, in linear (a, c) and semi-log (b, d) scale.

Table S2. UiOs BET surface areas and pore volumes calculated from the nitrogen and argon isotherms.

	BET areas (m^2/g)		***Pore volume (cm^3/g) @ p/p0=0.4****	
	N_2 (77K)	*Ar (87K)*	*N_2 (77K)*	*Ar (87K)*
UiO-66	1050	980	0.46	0.40
UiO-66_F	1970	1960	0.74	0.67
UiO-66_A	1450	1360	0.60	0.52
UiO-67	2410	2380	0.96	0.85

*Pores smaller than appr. 3.5 nm

S3. Physicochemical Properties of MMMs

S3.1 Scanning Electron Microscopy (SEM)

To further assess the distribution of MOF particles within the polymer matrix, cross-sectional imaging of the Pebax/20 wt% UiO-66_F membrane was performed using both energy-dispersive X-ray spectroscopy (EDS) elemental mapping (C, O, Zr) and backscattered electron (BSE) imaging (Fig. S9). The Zr elemental map obtained from EDS and the BSE image indicate a homogeneous distribution of the UiO-66_F filler within the matrix. These findings are consistent with the cross-sectional SEM observations presented in the main text and Fig. 3, and support our interpretation that improved filler dispersion contributes to the enhanced gas separation performance of this membrane.

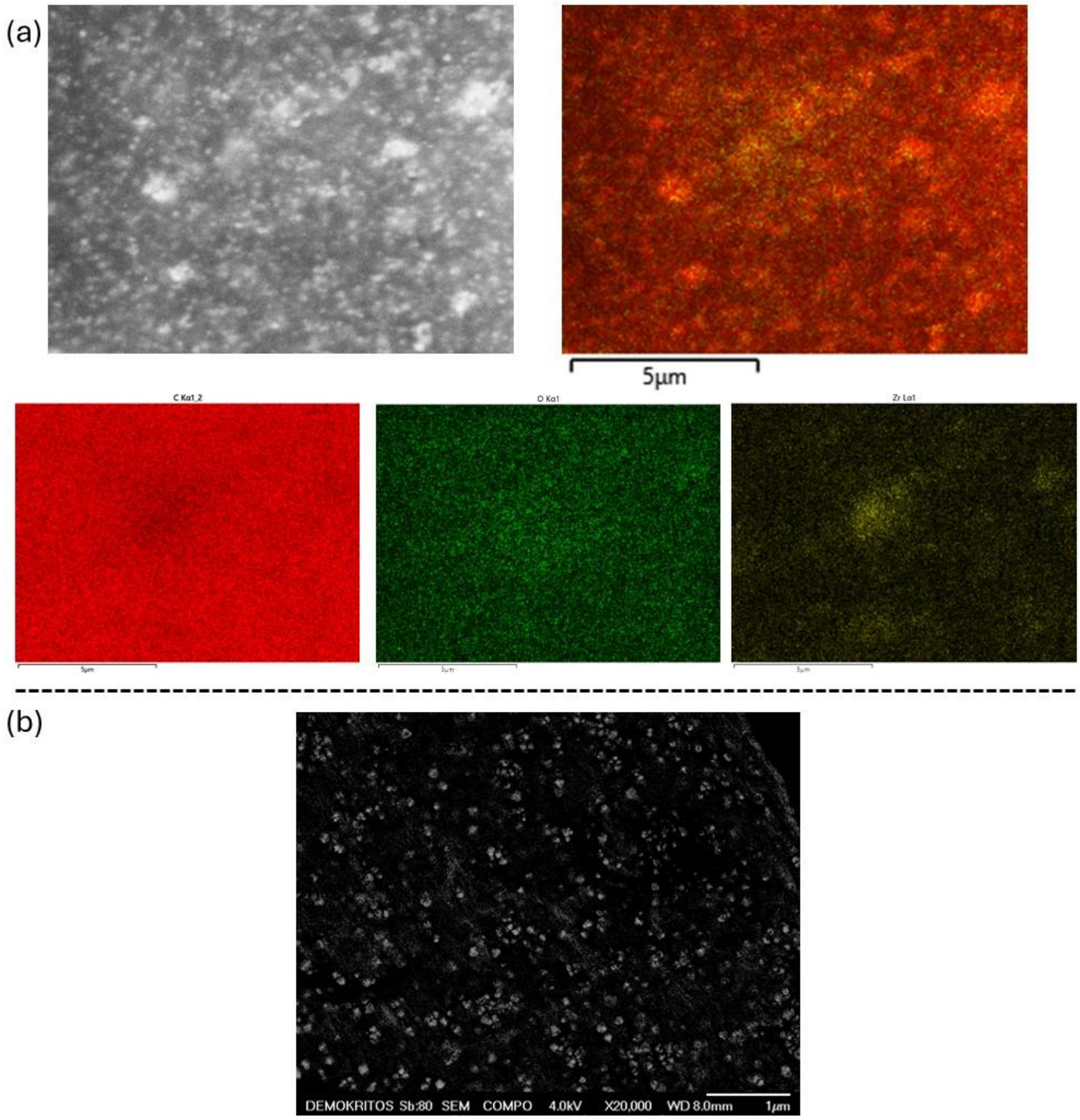

Figure S9. EDS elemental mapping distributions of C, O and Zr (a) and backscattered electron (b) SEM image of the Pebax/20 wt% UiO-66_F membrane cross-section.

S3.2 Thermogravimetric Analysis (TGA)

The actual MOF loading in each mixed matrix membrane was determined through TG analysis. At high temperatures (>550°C) a small residue (0.58% of the initial weight) was detected for the bare membrane This residue increases with MOF concentration in the polymer matrix due to the formation of ZrO_2 during the thermal decomposition process (see insets in Fig. 4). By considering the % mass retained after thermal decomposition for each UiO-type MOF when measured independently, the actual MOF content in the MMM can be calculated using the following equation:

$$\%Loading = \frac{m_{MMM} - m_P}{m_{MOF}} \times 100 \qquad \text{(S3)}$$

where m_{MMM} is the residual mass of each MMM, m_P the residual mass of the bare membrane (0.58%) and m_{MOF} the residual mass of the MOF as a standalone material (44.80% for UiO-66, 47.64% for UiO-66_F, 45.05% for UiO-66_A, 36.10% for UiO-67). The calculated actual MOF loadings are summarized in Table S3.

Table S3. Theoretical and actual MOF loadings of MMMs.

	**UiO-66**	**UiO-66_F**	**UiO-66_A**	**UiO-67**
**Loading (wt%)**				
**Theoretical**	**Actual**			
**5**	4.93	4.79	5.15	5.54
**10**	9.91	9.28	10.14	10.72
**20**	18.21	19.04	20.69	20.19

S4. Gas adsorption measurements - isosteric heat of adsorption

The CO_2 and CH_4 adsorption isotherms of the Zr-based MOFs measured up to 1 bar at 253, 263, 273 and 283 K are shown in Fig. S10. It is evident that all materials are selective to CO_2 over CH_4, as the uptake of CO_2 is consistently higher than that of CH_4 at the corresponding temperatures (e.g. 4-5 times higher at 1 bar for each temperature). The H_2 adsorption isotherms are not presented, as hydrogen adsorption capacity of UiO's is very low at temperatures near room temperature and pressures up to 1 bar, falling within the statistical error margin.

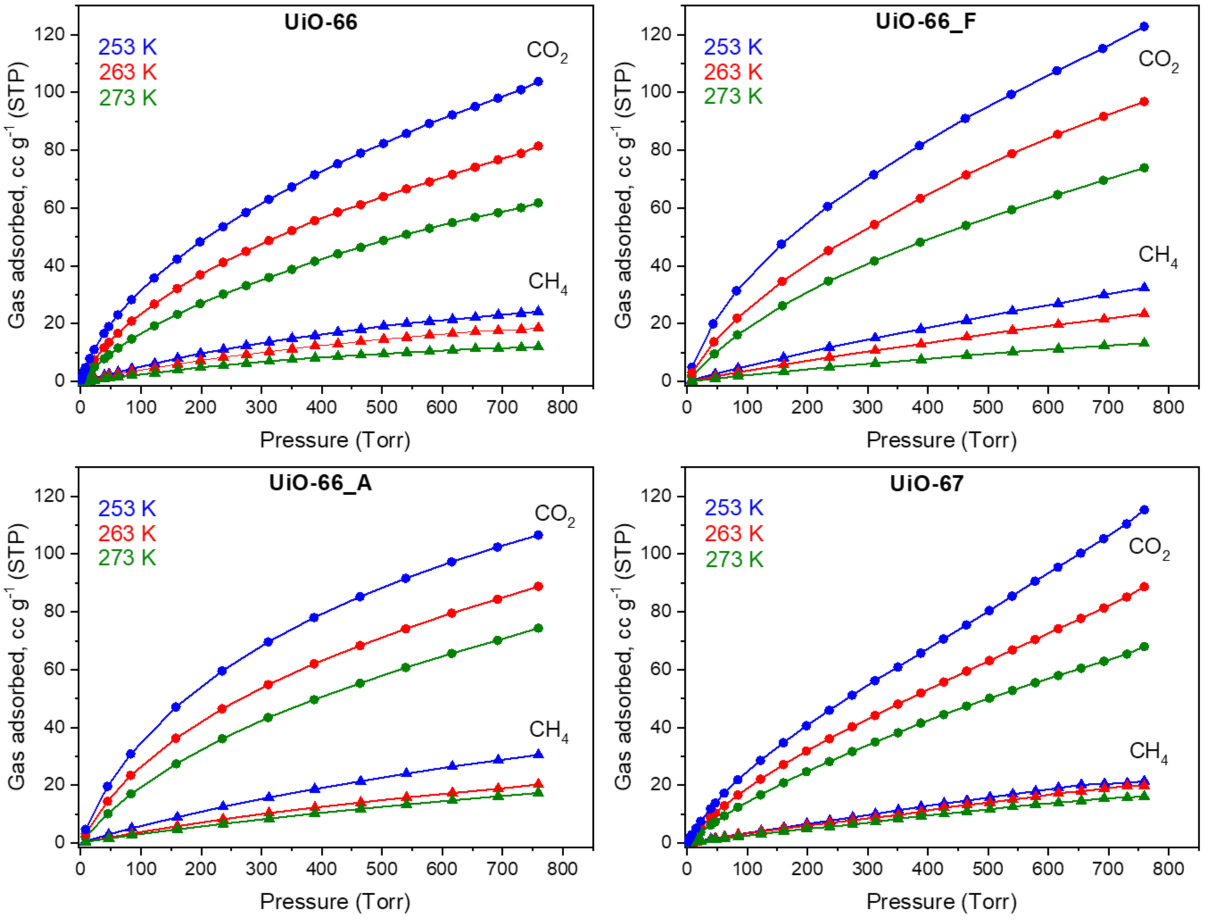

Figure S10. CO_2 (circles) and CH_4 (triangles) adsorption isotherms up to 1 bar recorded at the indicated temperatures (253K = blue, 263K = red, 273 = green).

Furthermore, the isosteric heat of adsorption (Q_{st}) for each UiO was calculated using the virial-type Eq. S4. The Q_{st} is a measure of the intensity of the interaction between the molecules of an adsorbed gas and the adsorbent's surface. More specifically, it describes the amount of heat released during the adsorption of gas molecules on the surface of the material as a function of the surface coverage.

The calculation of Q_{st} through a virial type equation requires the measurement of adsorption isotherms at different temperatures (typically three isotherms with a 10°C difference between

them). The data of the three isotherms are simultaneously fitted with the following equation:

$$lnP = lnN + \left(\frac{1}{T}\right)\sum_{i=0}^{m} a_i\, N^i + \sum_{i=0}^{n} b_i\, N^i \qquad \text{(S4)}$$

where, P is the equilibrium pressure, N is the amount adsorbed, T is the temperature, α_i and b_i are the virial coefficients and m, n are the numbers of terms required to sufficiently describe the isotherms. Finally, the heat of adsorption for zero coverage (Q_{st}°), which is practically the interaction energy of the first molecule that comes into contact with the surface of the material, and the heat of adsorption as a function of the amount of adsorbed phase are given by Eqs. S5 and S6, respectively:

$$Q_{st}^{0} = -Ra_0 \qquad \text{(S5)}$$

$$Q_{st}(N) = -R\sum_{i=0}^{m} a_i N^i \qquad \text{(S6)}$$

The CO_2 adsorption isotherms of all UiO-type MOFs at various temperatures up to 1 bar were fitted using the virial equation (Eq. S4) to calculate their Q_{st} as a function of CO_2 uptake. The CH_4 isosteric heats of adsorption were also calculated; however, due to the low CH_4 adsorption capacities of UiO-type MOFs, the results exhibited significant fluctuations. Consequently, they were not further analyzed or reported. As a representative example, the calculated Q_{st} of CO_2 for UiO-66_A is shown in Fig. S11. The virial coefficients obtained from the successful fitting of the isotherms (R^2=0.99974) are listed in Table S4.

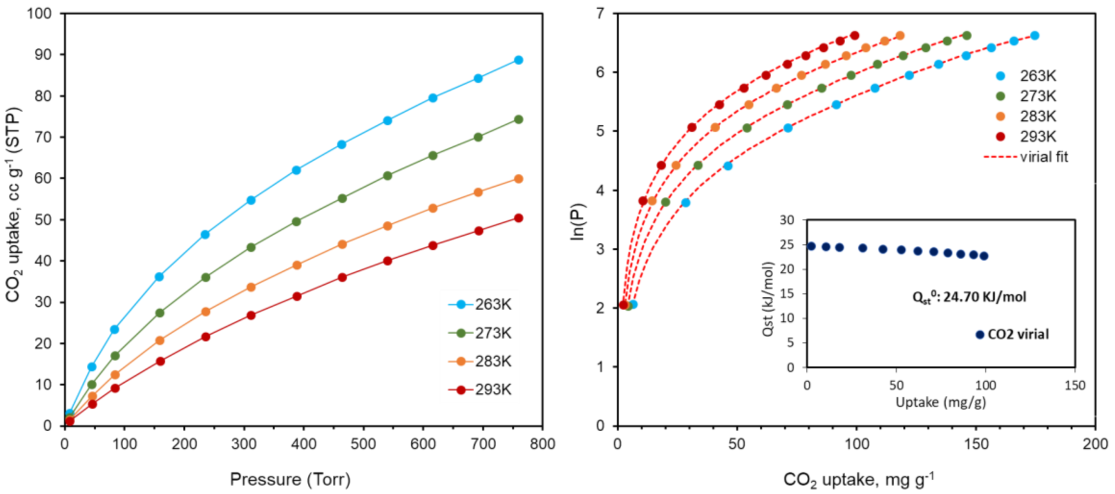

Figure S11. Left: CO_2 adsorption isotherms for UiO-66_A up to 1 bar at 263, 273, 283, and 293K. Right: Virial type fitting of CO_2 adsorption isotherms for UiO-66_A at 263-293 K, and the corresponding isosteric heat of adsorption (Q_{st}) as a function of CO_2 uptake (inset).

Table S4. Virial coefficient values for CO_2 obtained from the adsorption isotherms (up to 1 bar) fitting.

	**Virial equation**	
	**CO_2**	error
**a_0**	-2975.6243	70.28533
**a_1**	1.92458	2.48635
**a_2**	-0.01427	0.02051
**a_3**	0.00027	0.00007
**a_4**	-7.17×10^{-7}	2.07×10^{-7}
**b_0**	11.44132	0.25006
**b_1**	0.00802	0.00876
**b_2**	-0.00009	0.00007

The determined isosteric heats of adsorption as a function of CO_2 uptake for all UiO-type MOFs and the corresponding heats of adsorption at zero coverage ($Q_{st}°$) are illustrated in Fig. S12. These high values are consistent with published data, confirming the CO_2-philic nature of UiO-type MOFs. As anticipated, the denser, defect-free UiO-66 exhibits a higher affinity for CO_2 compared to the defective UiO-66_F and UiO-66_A, whereas the less dense UiO-67 shows the lowest affinity.

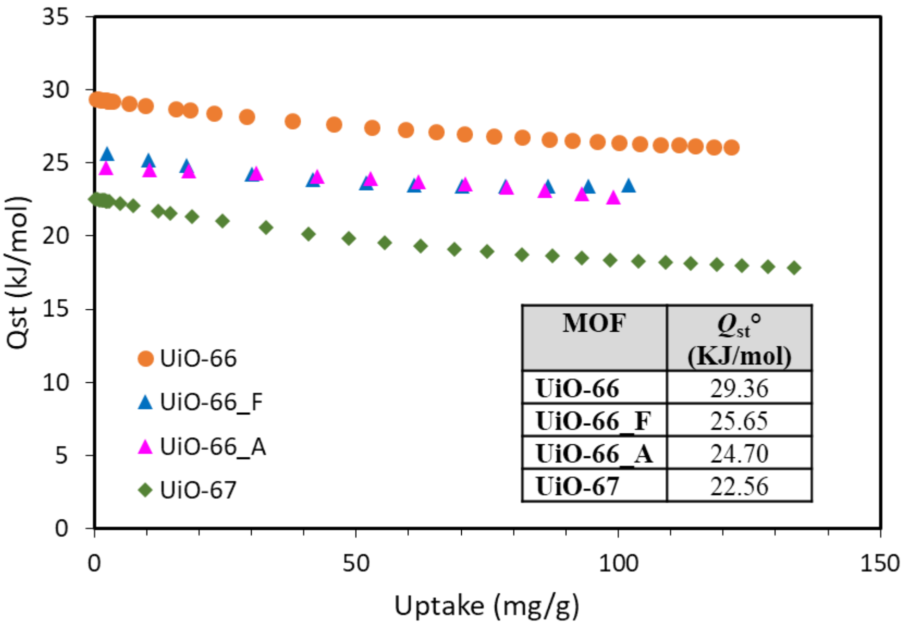

Figure S12. The isosteric heat of adsorption (Q_{st}) as a function of CO_2 uptake for UiO-66, UiO-66_F, UiO-66_A, and UiO-67 and the corresponding $Q_{st}°$ (inset table).

S5. References

[24] G. C. Shearer, S. Chavan, S. Bordiga, S. Svelle, U. Olsbye, K. P. Lillerud, *Chem. Mater.* **2016**, *28*, 3749–3761.

[57] L. Liu, Z. Chen, J. Wang, D. Zhang, Y. Zhu, S. Ling, K. W. Huang, Y. Belmabkhout, K. Adil, Y. Zhang, B. Slater, M. Eddaoudi, Y. Han, *Nat. Chem.* **2019**, *11*, 622–628.

[58] G. Kaur, S. Øien-ØDegaard, A. Lazzarini, S. M. Chavan, S. Bordiga, K. P. Lillerud, U. Olsbye, *Cryst. Growth Des.* **2019**, *19*, 4246–4251.

[59] M. Z. Ahmad, M. Navarro, M. Lhotka, B. Zornoza, C. Téllez, W. M. de Vos, N. E. Benes, N. M. Konnertz, T. Visser, R. Semino, G. Maurin, V. Fila, J. Coronas, *J. Memb. Sci.* **2018**, *558*, 64–77.
